# Advanced Signal-Amplification Strategies for Paper-Based Analytical Devices: A Comprehensive Review

**DOI:** 10.3390/biomedicines9050540

**Published:** 2021-05-12

**Authors:** Thi Xoan Hoang, Le Minh Tu Phan, Thuy Anh Thu Vo, Sungbo Cho

**Affiliations:** 1 Department of Life Science, Gachon University, Seongnam 13120, Gyeonggi-do, Korea; xoanht89@gmail.com (T.X.H.); vtathu0612@gmail.com (T.A.T.V.); 2 Department of Electronic Engineering, Gachon University, Seongnam 13120, Gyeonggi-do, Korea; 3 School of Medicine and Pharmacy, The University of Danang, Danang 550000, Vietnam; 4 Department of Health Sciences and Technology, GAIHST, Gachon University, Incheon 21999, Korea

**Keywords:** paper-based analytical devices, PADs, signal amplification, point-of-care diagnostics

## Abstract

Paper-based analytical devices (PADs) have emerged as a promising approach to point-of-care (POC) detection applications in biomedical and clinical diagnosis owing to their advantages, including cost-effectiveness, ease of use, and rapid responses as well as for being equipment-free, disposable, and user-friendly. However, the overall sensitivity of PADs still remains weak, posing a challenge for biosensing scientists exploiting them in clinical applications. This review comprehensively summarizes the current applicable potential of PADs, focusing on total signal-amplification strategies that have been applied widely in PADs involving colorimetry, luminescence, surface-enhanced Raman scattering, photoacoustic, photothermal, and photoelectrochemical methods as well as nucleic acid-mediated PAD modifications. The advances in signal-amplification strategies in terms of signal-enhancing principles, sensitivity, and time reactions are discussed in detail to provide an overview of these approaches to using PADs in biosensing applications. Furthermore, a comparison of these methods summarizes the potential for scientists to develop superior PADs. This review serves as a useful inside look at the current progress and prospective directions in using PADs for clinical diagnostics and provides a better source of reference for further investigations, as well as innovations, in the POC diagnostics field.

## 1. Introduction

Point-of-care (POC) diagnostics, which play an important role in personalized healthcare, have gained considerable attention owing to operations that enable medical staff to make rapid diagnoses and tailored treatment decisions. POC diagnostics offering affordable costs, easy operations without specially trained personnel, and rapid analytical consequences that have a positive correlation with the traditional clinical laboratory, are increasingly exploited in clinical diagnostic applications, especially if medical resources are limited [1,2,3,4]. Among the types of POC testing, paper-based analytical devices (PADs) have emerged as critical POC biosensors for disease monitoring and diagnostics, particularly in resource-constrained regions, for emergencies, and for in-house healthcare owing to their advantageous features, including simplicity, easy storage, disposability, and portability without relying on external devices [5,6,7,8]. There are three main classifications for PADs—lateral flow assays (LFAs), dipstick assays, and microfluidic PADs (µPADs) [9] with diversified signal readout approaches including colorimetry [10] luminescence [11,12] surface-enhanced Raman scattering (SERS) [13,14], photothermal methods [15,16], photoacoustic methods [17], and electrochemistry [18,19]. The most common detection approach in PADs is the colorimetric method, which can easily determine the presence of a target through color variations, for recognition even with the naked eye and without complicated instruments [20]. Despite the feasibility of PADs in POC diagnostics, comprehensive applications in diagnostic biosensors have still faced several challenges, such as low sensitivity and selectivity, poor quantitative discrimination, and limited stability [9,21], which have attracted research attention aimed at addressing these issues through signal-amplification methods.

**Figure 1:**
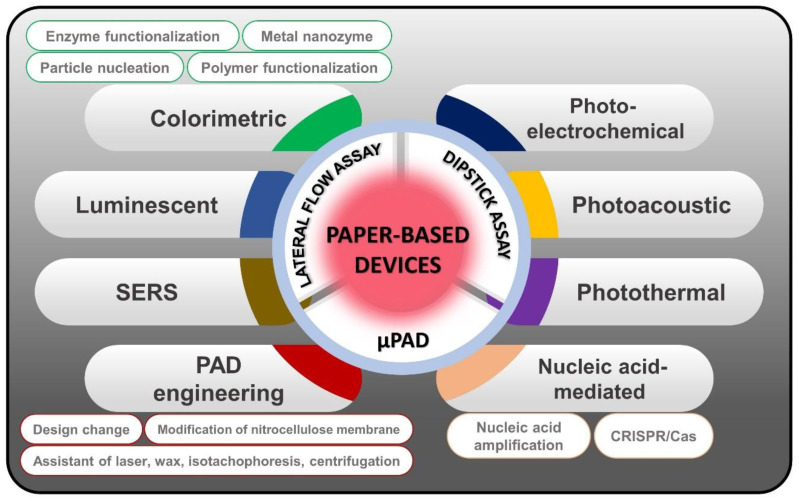
Signal-amplified paper-based analytical devices based on different enhancement strategies, including colorimetric, luminescent, SERS, photothermal, photoacoustic, photoelectrochemical, nucleic acid–mediated, and PAD engineering signal amplification.

In this review, we summarize the comprehensive signal-enhancement methods that have been developed to achieve superior PADs. These signal-enhancement methods, including colorimetric, luminescent, SERS, photothermal, photoacoustic, photoelectrochemical, nucleic acid-mediated, and PAD engineering signal amplification, are focused on in order to discuss nanostructures and sensing performance (Figure 1). We also highlight the utility of nanomaterials, owing to their outstanding properties in signal amplification. A comparison of different signal-enhancement approaches in terms of sensing capability is summarized to serve as an instruction set for scientists who want to develop superior PADs for use in fields ranging from scientific discovery to clinical applications.

## 2. Colorimetric Signal Amplification

Owing to its convenience and simplicity, the colorimetric lateral flow assay (CLFA) is one of the most prevalent diagnostic technologies for point-of-care applications, particularly for the detection of biomolecules [22,23,24], metal particulates [25,26,27], or food-contaminated pesticides [28,29,30]. In principle, colorimetric detection is associated with color development caused by an enzymatic or chemical reaction that can be seen with the naked eye or a semi-quantitative device. Nevertheless, it remains a great challenge to completely eliminate the background noise generated from the sample or the paper. Furthermore, due to the heterogeneity of the color distribution, evaluation of the final color is challenging. Such a limitation in sensitivity inhibits many critical applications, such as early detection of significant cancers and severe infectious diseases. With the rapid advancements in novel materials and nanotechnology, signal-amplification strategies that hold great potential to eliminate these limitations of CLFA have been developed in recent years. Significant effort has been dedicated to increasing light output yield, including controlling the physicochemical properties of the nanoparticle markers (such as metal label size and shape [31,32,33]), functionalizing nanocomposites with polymers [34,35], or employing enzyme-mimicking noble metal NPs [36,37,38,39].

### 2.1. Particle Aggregation and Size Enlargement via Nucleation

**Figure 2:**
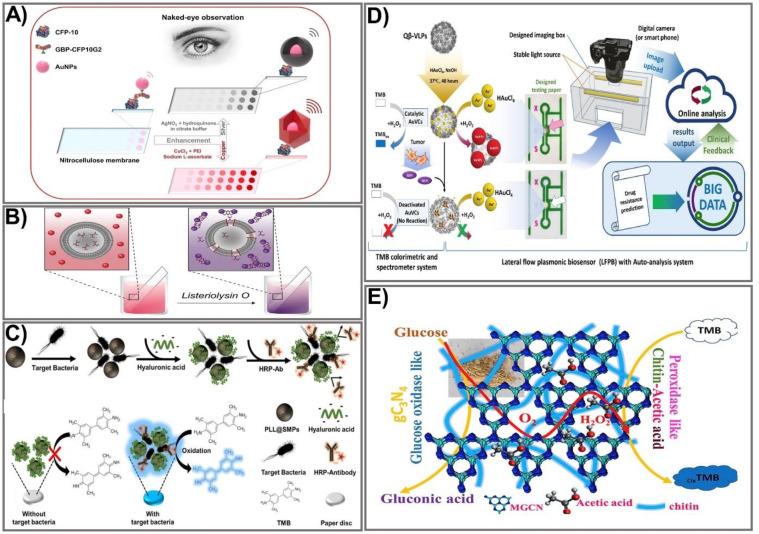
Strategies for signal amplification in colorimetric PADs. (**A**) A representation of naked-eye detection of CFP-10 using a copper/silver nanoshell enhancement-based dot-blot immunoassay platform. Adapted with permission from Ref. [48]. (**B**) The mechanism for detection of pore-forming toxin *Listeriolysin O (LLO)* using AuNP aggregation-based signal enhancement. Adapted with permission from Ref. [49]. (**C**) The design process and mechanism for detection of *Escherichia coli* O157:H7 bacteria using an enzyme functionalization-based signal enhancement strategy. Adapted with permission from Ref. [50]. (**D**) The principle of fabrication and detection of glutathione using peroxidase-like AuNP-based signal amplification. Adapted with permission from Ref. [51]. (**E**) The fabrication and detection procedure using a polymer-functionalized, metal-free dual enzyme-mimicking system to detect peroxide glucose. Adapted with permission from Ref. [37].

One of the most common and effective ways to enhance the colorimetric signal of paper-based devices is by enlarging the particle size. The plasmonic properties of noble metal nanoparticles make them a compelling candidate for developing colorimetric devices that can significantly improve output-signal intensity, thus enhancing the sensitivity to target biological molecules. Different types of NPs have been adopted for improved signal intensity, such as gold [40,41,42], silver [43], and copper [44]. Due to their unique optical properties and ease of preparation, gold nanoparticles (AuNPs) are the most attractive material for PAD fabrication. The most widely used platform includes the deposition of metal ions (such as gold, silver, and copper) on the surface of colloidal AuNPs, in which the AuNPs catalyze the surrounding metal ions into reduced atoms. This approach was applied by Rodriguez et al. for silver and gold enhancement-based lateral flow immunoassays [43]. The enhancement of AuNPs by Au ions is based on the reduction of Au ions on the surface of existing gold nanoprobes. In the presence of hydrogen peroxide (H_2_O_2_), chloroauric acid is a reduced gold ion, enabling the formation of a new gold layer on the surface of AuNPs [45]. The silver-enhancement process is similar in principle. In particular, silver lactate or acetate is usually used as the ion source to produce the Ag^+^ ion. Under the catalytic activity of AuNPs at room temperature, the Ag^+^ ion is reduced to form large agglomerates of metallic Ag in the presence of a reducing agent (such as hydroquinone). The general principle allows the formation of silver nanoshells around colloidal gold, making its color visible [43,46]. Silver- and gold-enhancement methods allow prostate specific antigen detection at a 0.1 ng/mL limit within 20 min. However, the uncontrollable Ag shell enhancement and the lack of specificity limit its broad application. To solve this problem, several metal nanoshell preparation methods have been designed. In particular, a well-controlled nanoshell formation process was developed by adding polyethyleneimine (PEI) to the reaction to act as the capping agent to obtain a shape-controllable nanostructure. The subsequent addition of sodium ascorbate (SA) leads to reduction of the forming complex, enabling the formation of a well-defined Cu nanopolyhedron shell that is quantitatively detectable with the naked eye [47]. This approach was successfully employed by Phan et al. to prepare Cu nanoshells on AuNP surfaces in dot-blot immunoassays for sensitive detection of the *Mycobacterium tuberculosis*–specific antigen CFP-10 [48]. The fabrication process is illustrated in Figure 2A. First, to prepare the paper-based immunoassay, AuNPs are integrated with the CFP-10-specific antibody GBP-CFP10G2 to form GBP-CFP10G2-AuNP conjugates. Then, the conjugates are immobilized on a nitrocellulose paper strip pre-coated with the CFP-10 antigen. When a solution containing Cu^2+^-PEI-SA is added to the paper strip, the antibody–antigen immunoreactions cause the formation of Cu nanoshells on the surface of the GBP-CFP10G2-AuNP. This preparation procedure for Cu nanoshells on AuNP surfaces not only results in the enlarged AuNP size but also leads to a particle shape change (from spherical to polyhedral) when Cu ions are attached to its surface, thereby significantly amplifying the signal intensity. The proposed platform enables detection of the *M. tuberculosis*-specific antigen CFP-10 with an LOD of 7.6 pg/mL, indicating approximately 13 times more sensitivity than another AuNP-based surface plasmon resonance method. The as-prepared platform was developed by the same group using gold and copper nanoshell enhancement for highly sensitive detection of Ag85B antigen with LODs of 1.56 and 0.75 ng/mL, respectively, for gold and copper enhancement visible with the naked eye, indicating more sensitivity than the silver enhancement process [10].

Owing to their ability to aggregate or disaggregate, AuNPs have also been extensively used for colorimetric immunoassays in which detection colors change from wine red to blue, an effect attributed to the agglomeration of AuNPs [52,53]. The assembly of aggregates allows an increase in the number of markers in the test zone, thereby improving the intensity of the assay. A simple and rapid sensing platform based on AuNP aggregation was reported by Mazur et al. for detection of *Listeriolysin O (LLO)* [49]. The design of the device was based on the formation of an *LLO* pore complex on cysteine-preloaded liposomes, causing the liposomes to release cysteine (Figure 2B). The freed cysteine then interacts with the AuNPs, leading to AuNP aggregation. Based on that principle, in the absence of *LLO,* intact liposomes and AuNPs do not interact with each other, causing the mixture to remain red. When *LLO* is present, AuNP aggregates formed by the interaction between released cysteine and AuNPs cause a colorimetric change from red-purple to blue, allowing for quantitative and qualitative detection of *LLO*. A cysteine-loaded liposome was deposited on the surface of Whatman filter paper. The moist paper was then immersed in a solution containing AuNPs. The colorimetric change elicited by AuNP aggregation in the presence of *LLO* enables *LLO* detection from as little as 12.9 µg mL^−1^ in PBS and 19.5 µg mL^−1^ in spiked human serum within 5 min, for an 18-fold enhancement in sensitivity compared to other liposome-based *LLO* detection assays [54]. An alternative way to prepare AuNP aggregates is based on a high concentration of salt in the reaction solution. Such a preparation method was employed by Diaz-Amaya’s group [55]. Taking advantage of salt concentration-induced AuNP aggregation, a highly stable multilayered label particle (ssDNA-PEI-Au-PS) was designed for multiple detection of mercury and arsenic. By employing PEI as an enveloping agent and using DNA aptamers as capture biomolecules, a sensitive colorimetric response was achieved in a controllable fashion. The proposed paper-based platform allows detection of mercury and arsenic at levels as low as 1 ppm in DI water and 2 ppm in river water. Other nanocomposites fabricated by conjugating different materials have also been exploited to take full advantage of the aggregation/anti-aggregation mechanism. Basiri et al. proposed an innovative and facile paper-based colorimetric assay based on AuNP-immobilized reduced graphene oxide (rGO@AuNP) nanocomposites for sensitive detection of dopamine and Cu^2+^ ions in human urine and tomato samples, with the detection limit reduced to 16 nM and 9.8 nM for dopamine and Cu^2+^, respectively [56]. Another nanocomposite, hemin-graphene nanomaterial (H-GN), which possesses tailored dispersibility in a high salt concentration and a highly active biomimetic oxidation catalyst property, was used for fabrication of a label-free colorimetric sensor for telomerase activity. The major challenges with metal NP aggregation-based signal amplification are false positive signals or a high background resulting from NP auto-aggregation, which is caused by uncontrollable external factors (for example, salt concentration, pH level, or temperature). However, with the recent development of nanomaterials and nanotechnologies, the abovementioned issues are expected to be tackled in order to improve the use of colorimetric assays in paper-based devices.

### 2.2. Enzyme Functionalization

With the recent achievements in nanotechnology, the employment of enzymes is a promising approach to improving signal intensity due to their remarkable catalytic properties, easy miniaturization, and substrate specificity. The utilization of enzymes for signal amplification is based on the catalytic activity of a natural enzyme that causes oxidation or reduction of a substrate, which in turn leads to sequential activation of the downstream enzymes that result in synergistic signal amplification within seconds. Currently, many multi-enzyme cascade systems have been developed, allowing rapid and sensitive detection of targets by paper-based sensing devices [57,58,59,60,61]. Another way to intensify the enzyme-labeled signal is to functionalize enzymes by linking them with nanoparticles [62]. The most commonly available enzymes used for signal amplification include horseradish peroxidase (HRP), alkaline phosphatase (ALP), and β-galactosidase. Enzyme-based signal amplification comprises the following: (1) employment of multiple enzyme complexes to accelerate the sequential enzyme reaction [63,64], or (2) tailoring of enzymes on the paper surface by nano-bioconjugates or polymers, allowing stable and function-modifiable attachment of enzymes that can significantly amplify a signal intensity that is linear to the analyte concentration, allowing for a naked-eye readout or semi-quantification with a spectrometer. Among the most common HRP substrates used in commercial immunoassays is 3,3′,5,5′-tetramethylbenzidine (TMB). An HRP-conjugated antibody and a TMB system were employed for specific signal amplification in the presence of the highly virulent bacteria *Escherichia coli* O157:H7 in sausages [50]. The detection platform was fabricated using magnetic separation combined with HRP-mediated signal amplification on a paper disc. The fabrication process includes the synthesis of the magnetic particle by integrating a short-chain glucan (SCG) with iron oxide NPs pre-coated with dextran (Dex@IONPs) to form a starch magnetic particle (SMP). The SMP surface was then functionalized with poly-L-lysine into the PLL@SMP composite, which was then added to the sample solution containing bacteria at different concentrations, followed by incubation with HA. For specific capture of bacteria, an HRP-conjugated bacterial specific antibody was used. The detection areas were prepared by soaking the paper discs in the TMB solution and allowing them to dry. Finally, the TMB-coated paper discs were immersed into the antibody-captured bacterial solution, and color change upon the reaction of HRP and TMB detected the bacterial concentration. Hyaluronic acid was used as a blocking agent to increase sensitivity and minimize the background signal (Figure 2C). The capture efficiency of the synthesized PLL@SMP-based platform for pathogenic bacteria was calculated at higher than 90%. The proposed sensing system was successfully used to detect the target pathogenic bacteria *E. coli* O157:H7 in sausage samples with an exceptionally low LOD of 30.8 CFU/mL, an approximate 300-fold enhancement in comparison to the conventional paper-based detection platform [65].

Besides HRP, alkaline phosphatase, glucose oxidase (GOx), and β-galactosidase also show significantly enhanced colorimetric signals on sensing platforms. Notably, ALP has been employed as a signal-amplification enzyme in many colorimetric immunoassays, owing to enzymatic activity that catalyzes the dephosphorylation or transphosphorylation of phosphate molecules [66]. Another commonly employed enzyme is GOx, which is used for glucose-level assessment. GOx can catalyze the oxidation of glucose to produce H_2_O_2_, which in turn is the substrate of the next enzyme-catalyzed reaction. Recently, GOx has been coupled with other enzymes, such as HRP, to design colorimetric immunoassays with significantly improved color development [60,67]. The current progress in enzyme functionalization strategies has seen significant advances in developing new and sensitive enzyme-aided paper-based sensing devices, providing promising sensing devices for sensitive detection of various molecules.

### 2.3. Metal Nanozyme Modification

Due to their high catalytic activity and stability, nanostructured artificial enzymes known as nanozymes have been considered effective alternatives to natural enzymes, offering attractive advantages. Compared to natural enzymes, nanozymes possess distinct advantages, including robustness, high catalytic activity, low cost, good stability, and easy mass production [68,69]. A number of nanomaterials with various compositions and nanostructures have been successfully synthesized, and they possess highly catalytically enzyme-like activities, including oxidase-, peroxidase-, and catalase-like nanozymes. Various well-controlled bimetallic NP-based nanozyme composites have been successfully used to enhance signals by mimicking natural peroxidases when integrated into colorimetric PADs. In principle, nanozyme-based colorimetric assays are based on the catalytic activity of the nanozyme toward chromogenic substrates, giving rise to color change that is correlated with the target concentration. Among the various NPs, AuNPs have exhibited excellent enzyme-mimicking activity, including peroxidase- and GOx-like activity. The peroxidase-like activity of gold-viral biomineralized nanoclusters (AuVCs) has also been successfully exploited as a nanozyme for the design of a lateral flow plasmonic biosensor (LFPB) for on-site glutathione (GSH) determination visible to the naked eye that has been quantified by auto-analysis software (Figure 2D) [51]. Initially, bioproduction of Qβ virus-like particles (VLPs) was prepared via transformation of the Qβ coat protein genome (pCDF–QβCP) into *E. coli*, enabling self-assembly of the QβCP into VLPz, which was then incubated with AuNPs in the presence of HAuCl_4_ and NaOH to generate AuNCs. The as-prepared AuVCs exhibited a high capacity when used to catalyze the formation of AuNPs in the presence of HAuCl_4_ and H_2_O_2_. In the presence of increased target analyte GSH, the AuVCs were deactivated, causing decreased catalytic activity, and a resultant reduction of AuNP formation was achieved, allowing for determination of the GSH concentration. Testing paper that consisted of recognition lines and flow-detection zones was printed from a wax printer. The sample was captured by an image box with a smartphone or digital camera and sent to online auto-analyzing software. Under optimized conditions, grayscale values plotted against GSH concentrations exhibited a linear relationship within the range 25–500 µM (LOD 9.80 µM), which is about a six-fold improvement compared with another paper-based electroanalytical test strip [70], with a highly positive correlation between the detected GSH level and the temozolomide (TMZ) drug-resistance level in glioblastoma multiforme (GBM) cells. Being oxidase-like is another good activity exerted by metal oxides that have been employed for signal improvement in several paper-based devices. Taking advantage of oxidase-like degradable manganite nanowires (γ-MnOOH NWs), a facile colorimetric paper sensor using γ-MnOOH NWs as a degradable nanozyme and TMB as a chromogenic indicator was developed for rapid and sensitive screening of organophosphorus pesticides (OPs) and acetylcholinesterase (AChE) [71]. The sensing mechanism was based on the oxidase-like activity of γ-MnOOH NWs in the presence of TMB as a chromogenic substrate. The reaction of acetylcholinesterase–acetylthiocholine (AChE-ATCh) releases thiocholine (TCh) that subsequently causes the degradation of γ-MnOOH NWs into an invalid Mn(II) ion, which could be used as a recognition element for AChE inhibitors (OPs). Degradation of the γ-MnOOH nanozyme leads to a significant loss of enzymatic activity toward TMB oxidation, thereby reducing color development. Based on that mechanism, the concentration of OPs could be measured by the color change of the TMB product at absorbance wavelength 652. The as-prepared detection platform was integrated on test paper, achieving higher sensitivity compared to an acetylcholine-conjugated paper test strip [72]—LOD 0.1 mU mL^−1^ for AChE activity, 10 ng mL^−1^ for omethoate, and 3 ng mL^−1^ for dichlorvos in real serum and vegetable samples.

### 2.4. Conjugated Polymer Functionalization

Owing to their high biocompatibility, biodegradability, and chemical and environmental stability, polymers have been used for NP modification to improve mechanical, thermal, electronic, or optical properties. Recent advances have focused on the fabrication of flexible polymer nanocomposite-based sensing devices that have shown wide applicability with high sensitivity. Nanocomposites functionalized with polymers could greatly improve mechanical, physical, and optical properties as well as multi-functionality. In paper-based colorimetric detection platforms, the integration of a polymer with nanomaterials has been successfully used for significant signal improvement. Different types of polymers have been employed for paper-based sensor fabrication. Chitin, a natural polysaccharide, was recently used as a bridge between graphitic carbon nitride and acetic acid to obtain glucose oxidase and peroxidase mimics [37]. Taking advantage of glucose oxidase-like modified graphitic carbon nitride (MGCN) and peroxidase-like chitin–acetic acid (chitin-AcOH), Sengupta and co-workers developed an MGCN-chitin-AcOH nanocomposite in a paper test strip through immobilization of MGCN-chitin-AcOH and a TMB substrate using a polyvinyl alcohol hydrogel composite (Figure 2E). To achieve glucose oxidase-like activity, GCN was chemically modified by a calcination procedure. Subsequently, the hybrid MGCn-chitin-AcOH was prepared by a control fusion technique, allowing the formation of a biofunctional nanozyme. MGCN-chitin-AcOH, when in contact with glucose, oxidized glucose to gluconic acid and hydrogen peroxide, while chitin-AcOH decomposed the generated H_2_O_2_, as proved separately by concurrent oxidation of TMB. The developed method was successfully applied to detect H_2_O_2_ and glucose in human serum and urine with a low detection limit of 0.052 μM for H_2_O_2_ and 0.055 μM for glucose.

## 3. Luminescent Signal Amplification

**Figure 3:**
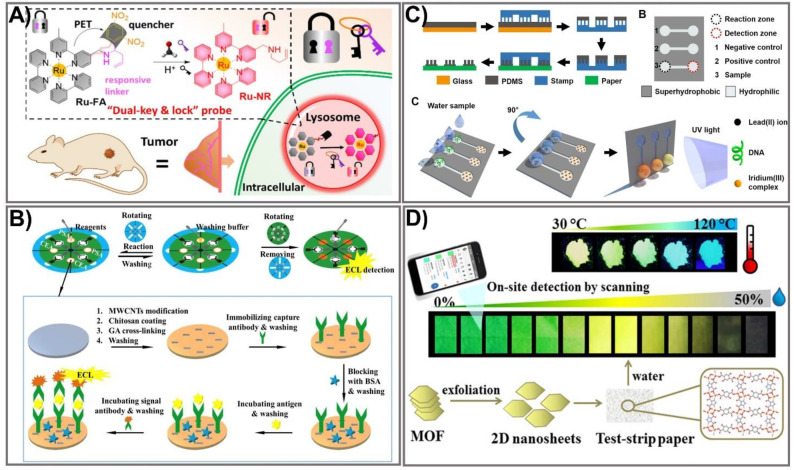
Luminescence-based PADs with enhanced detection sensitivity. (**A**) The schematic of the PAD assay for lysosomal formaldehyde detection using dual-key-and-lock ruthenium (II) complex probe as a signal amplification strategy. Adapted with permission from Ref. [77]. (**B**) Schematic illustration for the implementation of 3 paper discs in a rotational paper-based valve for a multi-step electrochemiluminescence immunoassay for the detection of carcinoembryonic antigen and prostate-specific antigen cancer markers. Adapted with permission from Ref. [79]. (**C**) Schematic representation of the suspended-droplet mode-based luminescence PAD design process for the detection of lead (II) ions in water samples. Adapted with permission from Ref. [81]. (**D**) The schematic principle of the excited-state proton transfer concept-based luminescence PAD for water and temperature sensing. Adapted with permission from Ref. [82].

Among the optical biosensors, luminescence-aided sensing, including chemiluminescence and bioluminescence, is a particularly compelling approach to signal transduction in many chemical sensing schemes due to the higher signal-to-noise ratio and the simplicity of the required measurement equipment. In general, detection with a luminescence-based device can be achieved by imaging or measuring the light emitted by bio-chemiluminescence or electro-generated chemiluminescence. Much effort has been dedicated to achieving an enhanced luminescence signal in PADs, mainly focusing on the choice of appropriate labeling agents, including the use of enzymes as labels in conjugation with enhancers to obtain improved signal intensity [73], metal-enhanced chemiluminescence [74], artificial pseudo-enzyme labels [75], and modified nanomaterial-based signal on-off mechanisms [76]. The use of nanomaterials has shown great promise in producing high-performance sensing devices due to their ability to act as the novel label of luminescent detection as well as being a platform to enhance the loading capacity of the luminescent labels. Liu’s group developed a novel dual-key-and-lock strategy-based ruthenium (II), or Ru(II), complex probe (Ru-FA) as an effective tool for formaldehyde detection in vitro and in vivo [77]. Ru-FA showed weak luminescence due to the photon-induced electron transfer (PET) process from the Ru(II) center to electron-withdrawing group 2,4-dinitrobenzene (DNB). Triggered by a specific reaction with formaldehyde (the first key) in an acidic environment (the second key), DNB is cleaved from Ru-FA, affording an emissive Ru(II) complex derivative, Ru-NR (Figure 3A). As a result of the PET process from the Ru(II) center to electron-withdrawing moiety DNB, Ru-FA itself displays weak luminescence, but its emission can be significantly increased after reacting with formaldehyde (key 1) in an acidic environment (key 2), accompanied by the production of emissive Ru-NR. This proposed system allows detection of formaldehyde at a 19.8 nM LOD, which is approximately 15 times higher than other paper-based analytical devices [78]. The on-off state of paper-based valves controlled by the rotation of paper discs is another strategy that has been used recently for improving a luminescence signal [79]. The rotational paper-based device was fabricated by assembling three designed paper discs using a hollow rivet. The on-off state of paper-based valves was easily controlled by rotation of the paper discs (Figure 3B). The integrated paper-based rotation valves can easily be controlled by rotating the paper discs manually, making it user-friendly for the untrained. In addition, the rotational valves are reusable, and the response time can be shortened to several seconds, which promotes the rotational paper-based device as offering great advantages in multi-step operations. Under the control of rotational valves, multi-step ECL immunoassays were conducted on a rotational device for multiplexed detection of carcinoembryonic antigen (CEA) and prostate specific antigen (PSA). The rotational device exhibited excellent analytical performance for CEA and PSA, which could be detected in linear ranges of 0.1–100 ng mL^−1^ and 0.1–50 ng mL^−1^ with limits as low as 0.07 ng mL^−1^ and 0.03 ng mL^−1^, respectively, which is approximately 10 times more sensitive than a paper-based fluorometric device [80]. Compared to other, conventional paper-based valves, the as-prepared platform could be dried for reuse, revealing its simplicity, rapidity, low cost, and excellent analytical performance. Due to its diverse structural polymorphism and an ability to switch on the signal upon binding to luminescence molecules, the non-canonical DNA secondary structure is another effective way to improve a luminescence signal. Sun et al. developed a paper-based µPAD based on a G-quadruplex-based luminescence switch-on assay for detection of the lead(II) ion (Pt^2+^) [81]. This type of suspended-droplet mode, paper-based µPAD uses wetting and gravity as a driving force. To fabricate the super-hydrophobic pattern on a paper device, a new microcontact printing-based method was applied by coating hydrophobic and transparent silicone polydimethylsiloxane (PDMS) on a glass slide attached with Teflon (a non-stick polymer allowing easy peel-off of the PDMS). For Pt^2+^ detection, G-quadruplex oligonucleotide and the iridium (III) probe, respectively, were added to the reaction zone and the detection zone of the test strip. The presence of Pt^2+^ allowed conformational change in the G-quadruplex, which can greatly enhance the luminescence emission of the iridium (III) probe (Figure 3C). The proposed platform was integrated into an inexpensive, battery-powered compact device for routine portable detection using a smartphone. Pt^2+^ was detected at low concentrations within the linear range from 10 nM to 100 nM. Excited-state proton transfer (ESPT) with huge luminescence Stoke shifts and an ultrafast response is another way to enhance the luminescence signal intensity and has generated great interest. Recently, an ESPT concept-based luminescence sensor was designed for discriminative detection via enol-keto tautomerism [82]. To improve the sensitivity, two-dimensional (2D) nanosheets of a metal-organic framework (MOF), Cd_2_(2,5-tpt)(4,5-idc)(H_2_O)_4_, were synthesized via top-down liquid ultrasonic exfoliation technology for sensing water in dimethylformamide (Figure 3D). This sensor can serve as a dual-sensing mechanism along with luminescence color change via shifted emission (green to yellow) in low water content and can be a turn-off method in high water content. Such a 2D nanosheet-sensing platform was applied to a paper test strip for ease of water detection with a rapid response (<30 s), long-term stability, pH stability, good reusability, high selectivity, broad-range detection (0–50% *v*/*v*), and a low LOD value (0.25% *v*/*v*) that is considerably lower than the conventional MOF-based sensing platform [83]. The recent improvements in materials and nanotechnologies have created new opportunities to further enhance luminescence signals through numerous strategies, including the use of proper labeling agents, a choice of fabrication process, or the use of an electrical energy supply. These approaches have shown great promise in producing handheld, paper-based devices with more sensitive detection of analytes using miniaturized devices. With a comprehensive understanding of the luminescent PAD, new generations of these sensing systems promise to solve the limitations of previously established devices, and could be employed in commercial applications.

## 4. Surface-Enhanced Raman Scattering Signal Amplification

SERS is a sensing technique that generates and amplifies inelastic light scattering of molecules when they are adsorbed on metals like gold, silver, and copper. This scattering signal could be enhanced (up to 10 orders of magnitude) by modulating the frequency of excitation light and the localized surface plasmon resonance (LSPR) of the metallic nanomaterials, relevant to the Stoke and/or anti-Stoke lines of molecules. In a SERS-based assay, specific tags made of nanostructures, and molecules with known Raman fingerprints, are the detection agents. Measuring the peak intensity of Raman molecules allows for quantification of particle and analyte concentrations. Consequently, SERS has become a powerful tool in diagnostics due to its rapid, sensitive, and multiplexed outcomes.

**Figure 4:**
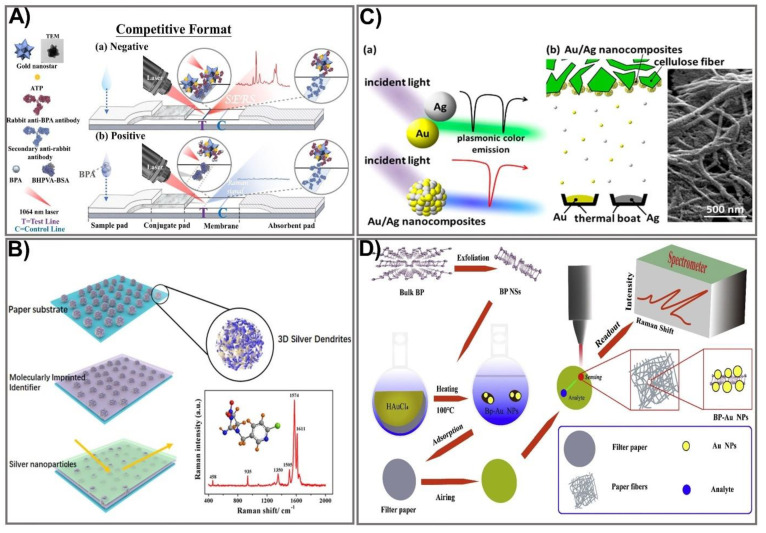
SERS-based signal amplification strategies for PADs. (**A**) The schematic principle of a gold nanostar-based SERS substrate for the sensing of biphenol A. Adapted with permission from Ref. [84]. (**B**) The design process for a multilayer SERS paper substrate for neonicotinoid pesticide sensing. Adapted with permission from Ref. [86]. (**C**) Schematic showing the design of a plasmonic alloy Au/Ag nanocomposite-based SERS substrate for signal enhancement in PAD-based biomarker sensing. Adapted with permission from Ref. [88]. (**D**) Representation of the design for a BP-Au filter paper-based SERS substrate for the detection of foodborne bacteria. Adapted with permission from Ref. [90].

Manipulating the shape, size, and composition of the nanostructure has shown great promise in improving the SERS signal. In particular, the gold nanostar (GNS) providing high SERS performance was used as a Raman reporter for PAD fabrication that showed significant signal intensity in the detection of bisphenol A (BPA) [84]. Gold nanostars with a sharp branched shape are great material for tuning plasmonic properties, providing the strongest SERS activities. GNSs were used as a SERS nanotag in comparison with spherical AuNPs. Both GNSs and AuNPs were tagged with the Raman reporter molecule ATP, followed by incubation with an anti-BPA antibody to form GNS/AuNP-antibody-ATP conjugates. BSA was subsequently used as a blocking agent, and 4,4-bis(4-hydroxyphenyl) valeric acid (BHPVA) was allowed to be bound by BSA to prepare the BHPVA-BSA conjugate. An LFA strip was used, consisting of a nitrocellulose membrane, a conjugate pad, an absorbent pad, a sample pad, and a support sheet. On the conjugated pad, the GNS/AuNP-ATP-antibody conjugate was dispersed, and the nitrocellulose membrane was coated with BHPVA-BSA and a goat anti-rabbit antibody as a control. For BPA detection, the BPA solution was dropped on the sample pad, where the analyte migrates to the membrane and conjugate pad, and finally moves to the absorbent pad. The color change in correlation with BPA concentration caused by the presence of GNS allowed quantitative detection of BPA (Figure 4A). The as-prepared SERS-LFA allows BPA detection at concentrations ranging from 0.05–60 ppt with a detection limit down to 0.073 ppt, indicating it is about 136 times more sensitive than the PEGylated AuNP-based LFA [85]. An alternative strategy for SERS enhancement is multilayer nanostructure fabrication that takes advantage of all employed compositions. As such, a SERS paper-based sensor was constructed by decorating paper chips with a sandwich structure of 3D silver dendrite (SD)/electropolymerization of molecular identifier (EMI)/silver nanoparticle (AgNP) (Figure 4B) [86]. The SD was synthesized on the surface of paper chips using a chemical reduction growth technique followed by immobilization of the EMI layer that serves as a specific recognizer. Subsequently, the core enhancement AgNP layer was integrated on top of the sensing chip. The as-prepared SERS paper chip was successfully applied for in situ detection of imidacloprid (IMI), showing ultra-high sensitivity with a detection limit of 0.02811 ng mL^−1^, 2000 times more sensitive than other paper-based organic–inorganic manganese (II) halide hybrid sensors [87]. This multiple SERS enhancement paper chip holds great potential for screening a variety of contaminants. The plasmonic alloy of different noble metals is another effective way of providing high-quality opportunities for tuning plasmon resonance. Taking advantage of the hotspot effect that creates an intense electromagnetic field near the plasmonic structure, the plasmonic alloy has been used for sensitive biosensor techniques such as SERS. Recently, a paper-based plasmonic substrate with a plasmonic alloy of Au/Ag nanocomposites on hierarchical cellulose micro-/nanofiber matrices for highly sensitive metal-enhanced fluorescence (MEF) and SERS biosensor applications was developed [88]. In the fabrication process, Au and Ag were deposited on the cellulose fibers at a low-temperature wafer level below 100 °C to avoid damage to the paper substrate and cellulose fibers (Figure 4C). The Au/Ag nanocomposite was thermally evaporated from the surface of the cellulose fiber. The integration of Au/Ag nanocomposites that act as a plasmonic alloy allows two distinct extinction peaks from Au and Ag to combine into a single peak. This paper-based plasmonic alloy substrate enables a two-fold increase in fluorescence signals and selective MEF signals, compared to Whatman chromatography paper. The proposed sensing device allows detection of folic acid at a picomolar level (LOD 1 pM), which is 1000 times more sensitive than other carbon dot-based paper devices (0.28 µmol/L) [89]. Paper-based devices have also been fabricated for the detection of foodborne bacteria. However, the low intensity of conventional Raman spectroscopy and fluorescence interference makes it difficult to detect biological samples in the complex. One strategy to deal with this challenge is to develop three-dimensional (3D) SERS substrates that offer a larger surface area for absorbing more probe molecules, and that generate more hotspots for analyte binding. Such an idea was applied to developing a novel, label-free, filter paper-based 3D-SERS detection platform to detect and classify common foodborne bacteria, including *E. coli*, *Listeria monocytogenes*, and *Staphylococcus aureus* [90]. Black phosphorus-Au (BP-Au) nanosheets were used as a SERS substrate that could generate abundant hot spots, showing great potential to serve as excellent SERS substrates (Figure 4D). The BP-Au sheets were prepared by conjugating AuNPs on prepared BP sheets to form BP-Au sheets. Subsequently, the BP-Au sheets were applied to the filter paper surface. This BP-Au filter paper-based 3D-SERS substrate exhibited remarkably improved Raman signals, allowing for specific recognition and classification of three types of target bacteria at concentrations as low as 10^−7^ CFU/mL, with an enhancement factor of 2.4 × 10^4^ (17 times higher than the Au/Ag paper substrate) [91]. This paper-based sensing device would be beneficial in practical applications for food safety. The employment of enzyme-like noble metal NPs as substrates for SERS is another approach that significantly improves SERS signals. Taking advantage of nanozymes for the catalytic degradation of methyl mercury, several paper-based SERS sensing devices using nanozymes have been developed. In particular, Liu et al. reported fabrication of Au-NiFe-layered double hydroxide (LDH)/rGO nanocomposites, which are not only efficient nanozymes with oxidase-like activity but also efficient SERS substrates to determine organic mercury [92]. Under the free radicals of electron paramagnetic resonance (EPR) spectra and the binding energy of an X-ray photoelectron spectrometer (XPS), upon the oxidase-like catalytic reaction, the oxygen molecules are captured, leading to increased O_2_ radicals, while MeHg is degraded, resulting in the release of CH_3_ radicals. The as-prepared Au-NiFe LDH/rGO nanocomposite could be used to effectively degrade and remove 99.9% of organic mercury in two h without secondary pollution of Hg^2+^, and a low concentration of MeHg was detected (as low as LOD 1 × 10^−8^ M), which is 10 times more sensitive than the conventional paper-based device [93]. To date, much effort has been devoted to enhancing the signal output for SERS-based PADs. The innovative achievements in the bioscience and nanomaterial science fields hold great promise for boosting fabrication of highly sensitive, low-cost, and miniaturized devices for point-of-care applications.

## 5. Photothermal Signal Amplification

**Figure 5:**
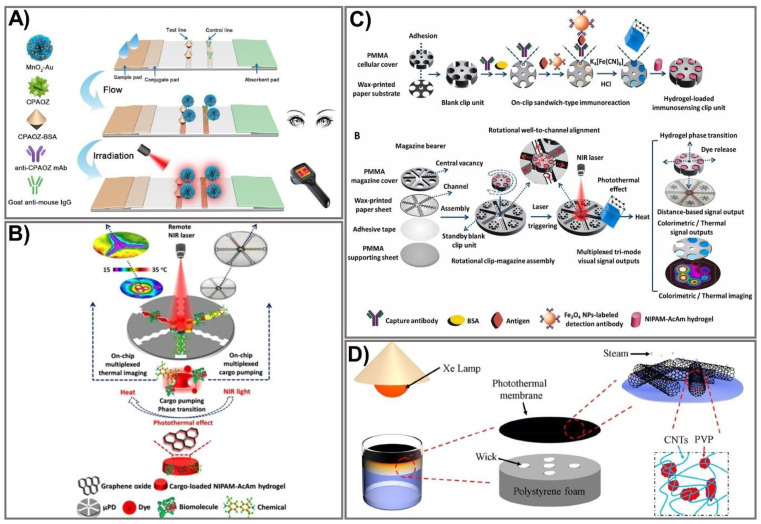
Schematic design of an amplified PAD signal using photothermal-based techniques. (**A**) Schematic representation of the MnO_2_-Au-based dual-signal immunoassay for AOZ detection. Adapted with permission from Ref. [100]. (**B**) Principle of photothermal microfluidic pumping-based PAD fabrication. Adapted with permission from Ref. [102]. (**C**) The design of the clip-magazine-assembled photothermal biosensing disk for tri-mode visual quantification of prostate-specific antigen. Adapted with permission from Ref. [103]. (**D**) Schematic demonstration of CNT@PVP photothermal membrane-based solar steam-generation device fabrication for water desalination. Adapted with permission from Ref. [104].

The photothermal activity of nanomaterials is another exciting property that has been exploited in recent years for the enhancement of paper-based sensing assays. Generally, the application of photothermal agents in the field of bioanalysis is based on light-to-heat conversion, which can be significantly intensified by using appropriate photothermal agents and a proper recognition system. Consequently, photothermal agent-mediated detection of a target can be carried out using a thermometer as a readout where the temperature signal is linear in relation to the target concentration. A wide variety of materials, including AuNPs [94], carbon NPs [95], quantum dots [96], and plasmonic NPs [97], have been exploited as photothermal agents for broad application in various fields. AuNPs that exert excellent photothermal signals have been extensively investigated to improve sensitivity [98,99]. Recently, a dual-signal biosensor based on multifunctional MnO_2_-Au was developed, enabling rapid achievement of qualitative information (visible to the naked eye) and quantitative photothermal data when detecting furazolidone antibiotic residues [100]. The fabrication process includes functionalization of AuNPs with MnO_2_ nanoflowers to form MnO_2_-Au signal probes that exhibit high colorimetric/photothermal properties. The MnO_2_-Au probe is then conjugated with a specific antibody (anti-CPAOZ mAb), followed by incubation with BSA to block non-specific binding. MnO_2_-Au exhibits a remarkably enhanced light-to-heat conversion effect attributed to the MnO_2_ nanoflowers’ high capacity to carry AuNPs. The flow immunoassay strip was composited from a nitrocellulose membrane, an absorbent pad, a sample pad, and a conjugate pad. Consequently, AOZ (the metabolite of FZD) can be quantitatively measured on the basis of both color change and heat signal (Figure 5A). The as-prepared immunoassay allows sensitive and comprehensive detection of FZD in food at low levels, attributed to the excellent light-to-heat conversion capacity of the MnO_2_-Au signal probe. FZD was detected at LOD 1 ng mL^−1^ and 0.43 ng mL^−1^ by colorimetric signal measurement and photothermal signal, respectively, indicating eight-fold improved sensitivity compared to the conventional colorimetric, gold-based lateral flow immunoassay [101]. The excellent performance exerted by this sensing device would be a promising sensing platform for broader application in point-of-care settings.

Recently, numerous advances in microfabrication techniques have been made, offering more facile and sensitive devices that possess many advantages over the conventional approaches. The microfluidic platform-based photothermal sensing assay is one of the currently emerging systems that hold great potential for paper-based point-of-care testing [102,105,106]. Microfluidic technologies have become increasingly powerful tools for developing point-of-care diagnostics because of their distinct advantages. However, the conventional microfluidic devices fabricated on silicon or glass surfaces require intricate design processes with complex instruments, limiting their practical application [107]. On the other hand, paper-based microfluidic devices exhibit great properties, including low cost, simplicity, sensitivity, and portability, making them a promising tool for point-of-care testing. The integration of the microfluidic platform with an intelligent sensing element is a novel strategy to compensate for the limitations of conventional microfluidic devices through synergistic performance in a single pattern. A photothermal microfluidic pumping approach was developed for an on-chip µPAD in a spatiotemporally controllable and contactless manner [102]. A multiplexed cargo reservoir fabricated from thermo-responsive poly(*N*-isopropylacrylamide)-acrylamide hydrogel-doped graphene oxide (GO) was immobilized on a paper surface (Figure 5B). The integration of composite hydrogels on highly photothermal-capable GO resulted in an on-chip phase transition of the composite hydrogels in a switch-like fashion. As a consequence, a strong pumping dynamic of the cargos from hydrogel to the reaction zones was achieved. By remotely controlling the laser power, GO density, and irritation time, the microfluidic pumping performance could be spatiotemporally manipulated. To demonstrate the efficiency of this pumping strategy, horseradish peroxidase and FeCl_3_ were used as the model cargoes to immobilize 3,3′,5,5′-tetramethylbenzidine and Prussian blue, respectively, as photothermal probes for µPAD-based colorimetric reactions. Owing to its high integrability in lab-on-chip paper-based devices, its controllable spatiotemporal capacity, and its features of being contactless and highly flexible, this novel microfluidic pumping platform shows great potential for numerous microfluidic applications.

Nevertheless, without the assistance of biological materials, photothermal sensing devices show remarkable limitations when applied to bioanalysis, including immunoassays. To overcome this challenge, Fu et al. developed a photothermal biosensing approach by integrating a photothermally responsive poly methyl methacrylate (PMMA)/paper hybrid disk (PT-Disk) in a clip magazine-assembled fashion [103]. The clip units consist of a honeycomb-patterned paper substrate and a hydrogel carrier fabricated from a PMMA cellulose cover conjugated with a specific antibody. These clip units were rotationally assembled on a magazine bearer composed of a strip-formed, channeled-paper sheet and an upper PMMA cover. To accelerate the photothermal conversion efficiency, Fe_3_O_4_ was captured on the paper substrate with an antibody and transported to a dual-functional probe—Prussian blue (PB) NPs that exert both colorimetric and photothermal activities. Subsequently, the dye-enveloped thermo-responsive hydrogels were loaded in clip wells. Upon laser irradiation, the photothermal effect of the PB NPs stimulated a rise in the dye solution’s temperature in a dose-dependent fashion, leading to the release of dye solution from the clip units to the surrounding magazine-bearing channels, which could be quantified by on-chip rulers and a portable camera (Figure 5C). The as-proposed sensing device allows analyte detection in a tri-mode signal output approach, including colorimetric readout on the paper disk for thermal-image and distance-reliable visual quantification. Using this novel sensing platform, the cancer biomarker (a prostate-specific antigen) was detected at LOD 1.4–2.8 ng mL^−1^, which was lower than the previously reported practical diagnostic threshold [108,109].

Due to their unique properties, photothermal-based devices have been employed for broad application in various fields, including controlling evaporation rates under solar irradiation. The hydrophobic surface is well-known for the design of water treatment systems. However, interfacial thermal resistance events occurring after hydrophobic treatment usually lead to remarkably low energy efficiency, limiting the water treatment process. To address this limitation, a carbon nanotube (CNT)-modified superhydrophobic photothermal membrane was recently fabricated on a filter paper substrate for effective water desalination upon solar irradiation [104]. In particular, the superhydrophobic photothermal membrane was composited from the CNT and polyvinylpyrrolidone (PVP) modified with 1H,1H,2H,2H-perfluorodecyl (CNT@PVP). The CNT@PVP composite was sprayed on a filter paper surface to produce a photothermal membrane that is impermeable to water. The membrane was placed onto an expanded polystyrene-prepared bottom stand for flexible floating and heat localization (Figure 5D). The as-prepared device exhibited high energy efficiency (91%) with an increased evaporation rate of 1.41 kg m^−2^ h^−1^, indicating higher desalination efficiency than previously established devices [110,111]. The key advantages of the photothermal conversion system include a high light-to-heat capability, reusability, stability, low cost, and portability, making it a promising tool for signal amplification and quantification of PADs.

## 6. Photoacoustic Signal Amplification

**Figure 6:**
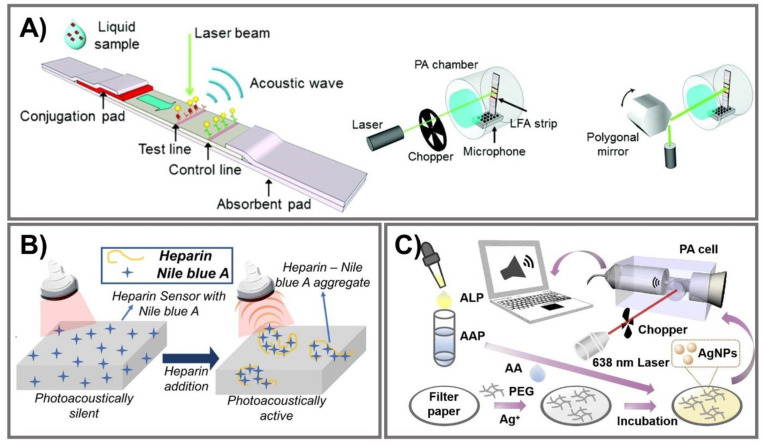
Photoacoustic signal amplification. (**A**) From left to right, PA-based LFA strip illuminated by a laser beam to generate PA signals; illustration of PA measurement systems for chop mode and scan mode. Adapted with permission from Ref. [112]. (**B**) Illustration of a heparin sensor via interactions with Nile blue A to activate photoacoustic signals. Adapted with permission from Ref. [113]. (**C**) PA device for ALP detection via PA signal generation of AgNPs from hydrolyzed AAP on filter paper. Adapted with permission from Ref. [114].

Photoacoustic (PA)-enhanced signals have been developed in a few PAD-based biosensing platforms for cryptococcal antigen [112], heparin [113], and ALP [114]. Photon energy, strongly absorbed by chromophores, is converted into mechanical energy via thermal expansion, resulting in the production of acoustic waves as PA signals [115,116]. Zhao et al. developed photoacoustic-based LFA using AuNPs to quantitatively measure cryptococcal antigen (Figure 6A). Compared to semi-quantitative colorimetric analysis, the PA detection method improves the sensitivity of LFAs owing to the strong LSPR of AuNPs and the effective elimination of a PA background signal associated with ambient light noise. Light absorption was specifically designed to enhance the PA signal with effective minimization of the background signal from paper substrate. To confirm the enhanced effect of the PA method on LFA, the limit on detections measuring cryptococcal antigen was calculated for three different measurement methods, including colorimetric measurements (1.1 ng/mL), chop mode PA detection (0.57 ng/mL), and scan mode PA detection (0.010 ng/mL), indicating the capability of the PA method to decrease LOD by more than 100 times. This PA-based LFA offers advantages in terms of high sensitivity, reduced system noise, strong and reliable PA signal generation from AuNPs, and minimization of expensive photodetectors and optical filters [112]. Another paper-based photoacoustic sensor for direct detection of heparin was fabricated for point-of-care measurement of heparin activity in human blood samples via fingerprick-sized blood samples (Figure 6B). A cellulose-based photoacoustic heparin sensor was fabricated by loading cationic dye (Nile blue A) onto polyethylene glycol (PEG)-modified Whatman filter paper. Heparin, a negative-charged polysulfated glycosaminoglycan, can interact with Nile blue A to exhibit PA spectral alternation. This PA-based sensor showed LOD at 0.28 U/mL of heparin in human plasma with a turnaround time of 3 min and at 0.29 U/mL in whole blood within 6 min. The correlation of heparin, turnaround time, and sample requirement is comparable to an activated clotting time test. This PA sensor for heparin can monitor not only heparin concentration but also heparin activity in human serum samples, which is exploited as an affordable and disposable heparin sensor [113]. Instead of AuNPs, Zhang et al. developed a portable photoacoustic device for determination of ALP in serum using silver nanoparticles with excellent photo-stability and reproducibility (Figure 6C). The breakdown of sodium L-ascorbyl-2-phosphate into ascorbic acid was catalyzed by the ALP enzyme, thereby converting AgNO3 into AgNPs. Under irradiation from a modulated 638 nm laser, a strong PA signal generated by AgNPs due to their localized plasmon resonance was detected by the portable PA device. After optimizing the reaction condition, this PA-based sensor could detect ALP in a concentration range of 5–70 U/L with LOD of 1.1 U/L. This rapid portable PA device exhibits favorable reliability and stability for determination of ALP in human serum with high sensitivity, providing a PA-based analytical strategy to detect disease-correlated biomarkers [114]. Although the reproducibility of PA-based PADs should be thoroughly validated due to the intrinsic point-scanning reading of the PA technique, the PA technique may exhibit promising potential to measure biomarkers in clinical realities.

## 7. Photoelectrochemical Signal Amplification

Photoelectrochemical (PEC) detection has drawn increasing attention due to its promising analytical features, including low cost, simple equipment, portability, persuasive selectivity and sensitivity, and low background, facilitating high-throughput and quick assays [117,118,119,120]. Driven by these advantages, paper-based PEC systems that integrate the inherited excellence of cellulose paper and PEC bioanalysis have been fabricated [117]. The PEC platform stems from the electrochemical ideal, which can convert light energy into chemical and electrical energy, hence ameliorating sensitive detection through the modification of photocurrent responses into photoelectrochemical reactions based on analytes and specific photoactive matrix/probe interactions [117,121,122]. Photoactive electrons can be stimulated upon excitation by light, and conveyed to an electrode to generate a vigorous photocurrent signal, for which a signal-off or signal-on readout method is employed [117,122].

**Figure 7:**
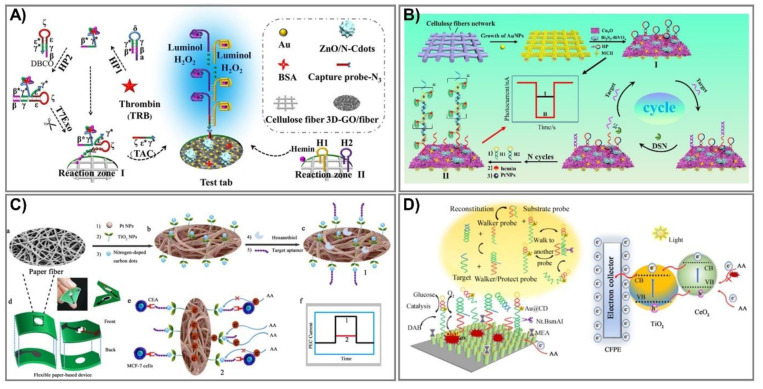
Photoelectrochemical paper-based analytical devices. (**A**) The intrinsic light Au@3D-rGO/cellulose sensor generates a photocurrent signal to detect protein in clinical biomedicine. Adapted with permission from Ref. [142]. (**B**) The construction process and mechanism for cathode PEC PADs shown to detect microRNA-141 by duplex-specific-nuclease (DSN) connection. Adapted with permission from Ref. [143]. (**C**) The PEC TiO_2_–Pt/PWE biosensor was fabricated by modification of paper with N-carbon dots/TiO_2_–Pt with a seed-mediated growth method to detect carcinoembryonic antigen (CEA). (a–c) preparation of photoelectrochemical biosensor, (d) diagram of paper-based platform, (e) detection mechanism, (f) photocurrent intensity with/without CEA. Adapted with permission from Ref. [144]. (**D**) An ultrasensitive PEC paper-based device to determine miRNA-141 from a two-enzyme-engineered DNA walker and a TiO_2_/CeO_2_ heterojunction. Adapted with permission from Ref. [128].

Despite outstanding achievements that have been obtained in lab-on-paper analytical devices, certain challenges for employed electrodes in assisting paper-based devices still exist. First, the most serious reason is associated with conductive inks that only cover the paper’s surface and are not assembled into the interior structures, leading to limited conductivity and, eventually, considerable influence on the sensitivity of the devices. The next problem is associated with practical stability under harsh conditions due to too-weak interactions between cellulose fibers and conductive materials. Finally, highly efficient lab-on-paper analytical devices urgently require remarkable capacity to generate, transmit, and measure electrical signals [123]. Therefore, PEC-based signal amplification enhancement that can address these issues is necessary in order to minimize the number of assays, but still reach high sensitivity with a very low concentration. In the last few years, diverse strategies have been proposed to amplify photoactive signals for PEC-sensing fabrication, most of which have been employed in electron acceptor/donor regulation [120,123,124,125,126], photoactive materials, nanozymes [119,120,127,128,129,130,131,132,133,134,135], or light sources [136,137,138,139,140,141,142]. Among them, functional nanomaterial integration is an emerging potent route to reach giant, efficient lab-on-paper analytical devices, and diverse photoactive nanomaterials have been immobilized on cellulose fibers. Although it is well known that an excited light source is requisite for a photoelectrochemical platform, physical light source-related instruments are also required [142]. Therefore, to achieve instrument miniaturization as well as operational simplification, luminol-based chemiluminescence emission was exploited as an interior light source, which offers multitudinous advantages, including low cost, simplicity, high sensitivity, and a rapid response [136]. Lan et al. designed and constructed a novel 3D-rGO/cellulose-based photoelectrical lab-on-paper analytical device to determine thrombin (TRB) in serum samples, a procedure typically utilized for Alzheimer disease and cardiovascular disease diagnosis [142]. The combination of reduced graphene oxide and Au flower in paper cellulose fiber facilitates superior conductivity as well as biocompatibility. ZnO anchored/nitrogen-doped carbon dots are immobilized in this system and stimulated by strong chemiluminescence of a luminol–H_2_O_2_ system to generate a photocurrent signal (Figure 7A). This proposed sensing performed with excellent sensitivity, as well as specificity in thrombin determination, and can detect very low concentrations of 16.7 fM.

Alternatively, sensing can obtain the dual functions of target recognition and signal transduction, which are widely employed in cyto-assays due to their triggering of the signal of target recognition [123]. Recently, the electron acceptor/donor methodology has been widely investigated for enzyme-catalyzed consumption/generation, which are exerted as co-reactants in hole-oxidization reactions and electron moderation, leading to cathodic/anodic photocurrent signal production [143]. Li et al. fabricated a novel sensing stand on the typical catalytic reaction of a hemin/platinum nanoparticle (Pt NP) trunk-branching-decorated DNA dendrimer toward H_2_O_2_ [143]. Pt NPs have been widely exerted in bioassays due to their excellent inherited properties involving small size, diverse enzyme-mimic activities, and especially catalytic activity toward H_2_O_2_ for electron acceptor (O_2_) production [145,146]. Thus, in this proposed sensor, the DNA dendrimer serves as enzyme-like activity enhancement and synergy catalysis with Pt NPs, as well as hemin, to catalyze H_2_O_2_ and increase electron acceptors; hence, it can determine the target miRNA-141 over a wide range, from 0.5 fM to 5 nM with a limit of detection at 0.17 fM (Figure 7B).

In photoactive material-associated PEC paper-based devices, photoactive species play an essential role serving as a light absorber [119]. In particular, emerging nanozymes are being intensively investigated as an expected alternative to natural enzymes due to their many intrinsic qualities, including superior stability, enzyme-like capabilities, and wide pH operating range [128]. For instance, Li and coworkers fabricated photoelectrochemical paper-based sensing to detect carcinoembryonic antigen (CEA) based on the modification of paper with N-carbon dots/TiO_2_–Pt in a seed-mediated growth method [144]. First, TiO_2_ seeds were prepared from a TiCl_3_ solution in a mixture maintained at 80 °C for 2 h and then dried at 450 °C for 2 h. In the next step, these TiO_2_ seeds were spread onto cellulose fiber (Pt/PWE) and equilibrated at 37 °C, repeating the process three times. The proposed TiO_2_ NP-modified photoelectrochemical lab-on-paper analytical devices can determine CEA concentrations based on the reduction of the photoelectrochemical signal, because they bind with CEA at the MCF-7 cell surfaces in human serum (Figure 7C). This novel platform has good biocompatibility, is portable, and has eco-friendly devices that can detect low levels in limitations of 1.0 pg mL^–1^ over a broad linear range of 0.002–200 ng mL^–1^. Additionally, Li and colleagues also employed this strategy to develop an ultrasensitive PEC paper-based device to determine miRNA-141 from a two-enzyme-engineered DNA walker and a TiO_2_/CeO_2_ heterojunction (Figure 7D) [128]. During cancer development, miRNA-141 is expressed in large proportions, and thus, acts as a potential marker for cancer diagnostics and assessment. In the presence of miRNA-141, the protecting probes would be kept away, leading to activated walker probes, creating an endonuclease cleavage reaction, and eventually, retrieving a signal output. The novel sensor can verify miRNA-141 in real human serum with LOD at 0.6 fM, and can ensure long-term stability and highly satisfactory selectivity.

## 8. Nucleic Acid-Mediated Signal Amplification

Aptamer-integrated PADs are emerging as potential diagnostic sensors due to the outstanding aptamer advantages of more reasonable costs, high thermo-stability, and non-toxicity, as well as fewer batch-to-batch variations in comparison to antibodies [147,148]. A paper-based nucleic acid amplification test has been developed with the support of nucleic acid amplification and clustered, regularly interspaced, short palindromic repeats (CRISPR)/Cas) systems to improve the sensitivity of PADs.

### 8.1. Nucleic Acid Amplification

Nucleic acid-associated signal amplification has attracted wide attention in the fabrication of highly sensitive paper-based point-of-care diagnostics employed for invading pathogens or viral determination due to the nucleic acid-amplification capacity. Especially during the current COVID-19 pandemic, the need for an innovative and effective methodology to detect the novel coronavirus (SARS-CoV-2) was evident in order to control, minimize, and finally prevent its spread. Conventionally, polymerase chain reaction (PCR) is the technique extensively used to rapidly process millions to billions of DNA samples, but it requires precise temperature control for reactions, and hence, to date has been unsuccessfully applied to PADs [149,150]. Alternatively, various signal amplification isothermal methods have been tried for PAD development, including rolling circle amplification (RCA) [147,151,152,153,154,155], loop-mediated isothermal amplification (LAMP) [156,157,158,159,160,161,162,163,164,165,166], strand-displacement amplification (SDA) [167], recombinase polymerase amplification (RPA) [168,169,170,171], helicase dependent amplification (HDA) [172,173,174], and nucleic acid sequence-based amplification (NASBA) [175].

**Figure 8:**
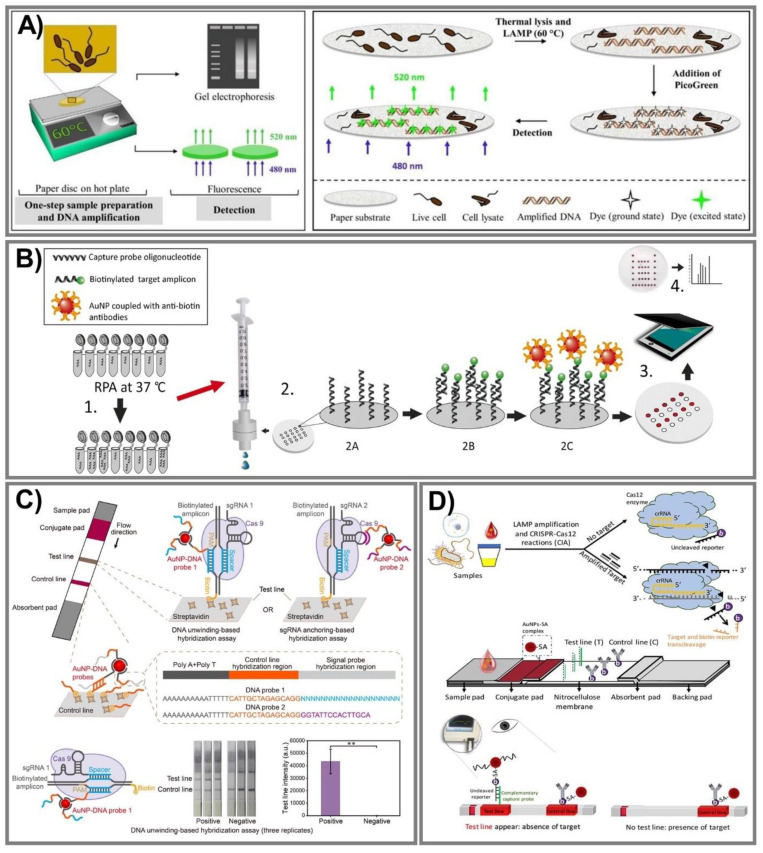
Nucleic acid-amplified PADs. (**A**) One-step lysis to amplify DNA for *E. coli* and *M. smegmatis* in a LAMP system through recorded fluorescence intensities by the binding of DNA with PicoGreen dye. Adapted with permission from Ref. [161]. (**B**) The process for human adenoviral detection was carried out by novel RPA vertical flow-paper microarray based on the visualization of anti-biotin antibody-conjugated AuNPs with positive spots. Adapted with permission from Ref. [170]. (**C**) The CRISPR/Cas9-mediated lateral flow nucleic acid assay was established to identify *L. monocytogenes* and African swine fever virus (ASFV) in swine serum samples. ** *p* < 0.01 (two-tailed Student’s *t* test). Adapted with permission from Ref. [177]. (**D**) An ultrasensitive CRISPR/Cas12 lateral flow sensor was developed by integrating Cas12a and Cas12b effectors to determine *Pseudomonas aeruginosa*—a multidrug-resistant infection. Adapted with permission from Ref. [178].

The principal difference in the aforementioned isothermal methods stems from primer annealing and extension, and LAMP, RCA, and RPA have been employed widely in PADs. In particular, the LAMP technique provides enlarged specificity and sensitivity based on an exponential amplification capacity that can distinguish primers in one reaction, and hence, can simultaneously identify various target sequences [176]. To determine a mycobacterium’s DNA in a single reaction for the first time, Naik and coworkers successfully designed a novel LAMP assay on a paper substrate to detect *E. coli* and *M. smegmatis* (Figure 8A) [161]. This system can perform lysis and amplify DNA from only 100 CFU/mL with high sensitivity within 30 min through recorded fluorescence intensities, especially ensuring safety due to an effective ability to kill all bacteria in the samples. On the other hand, in RCA, this isothermal DNA amplification method can simplify operations, producing 1000 complementary copies in linear concatenated DNA within only 1 h [151]. Bialy et al. constructed a one-pot-reaction paper-based POC device to detect thrombin and platelet-derived growth factor, typically through the suppression of RCA [152]. The results were obtained via the fluorescence signal of SYBR Gold dye and QuantiFluor dye with a very low LOD at 10 nm (3σ) and 25 nm (3σ), respectively, within 30 min at RT. In addition, RPA has been one of the most amplified methods for paper-based nucleic acid biosensor fabrication due to its outstanding ability to rapidly evaluate various gene targets at low temperature with superior primer specificity [170,171]. Based on these advantages, an RPA vertical flow paper microarray was developed for human adenoviral DNA detection by integrating it with colorimetric detection holding antibody-conjugated AuNPs (Figure 8B) [170]. This proposed system enables validation from 1 ng of starting material in clinical nasopharyngeal aspirate samples in less than 10 min for detection, and it is expected to be used for multiplexed viral diagnostics.

### 8.2. CRISPR/Cas Triggered Signal Amplification

Currently, the CRISPR/Cas effector is attracting a lot of attention because it is not only extensively employed in genome editing, but is also considered an innovative approach in biosensor fabrication. This system can offer much more precise (as well as rapid) analysis for ultra-sensitive nucleic acid evaluation. The CRISPR/Cas9 system has outstanding DNA recognition capacity, but does not provide trans-cleavage activity; hence, even though Cas9 conducted directed evolution with its genome-editing ability, it can still be harnessed to apply to biosensing system construction [177]. Furthermore, Cas12 and Cas13 recently emerged as potential strategies in which Cas12 can create a ternary complex with crRNA (or sgRNA) and a target nucleic acid, while Cas13 can be used against ssRNA, and then labeled with fluorescence or dye to generate fluorescence or colorimetric signals [179]. Various studies have reported excellent capacity in CRISPR/Cas systems, including Cas9 [177,180], Cas12 [178,181], and Cas13 [182]. Wang and coworkers established a CRISPR/Cas9-mediated lateral flow nucleic acid assay (CASLFA) to detect *L. monocytogenes* and the African swine fever virus, typically in swine serum samples (Figure 8C) [177]. This proposed system can recognize double-stranded DNA (dsDNA) with only hundreds of copies of genome samples in 1 h with an accuracy of 100%, in comparison to a real-time PCR assay. On the other hand, for Cas12 effector applications, an ultrasensitive POC CRISPR/Cas12 lateral flow sensor was developed by integrating *Lachnospiraceae bacterium* Cas12a and *Alicyclobacillus acidoterrestris* Cas12b effectors with the LAMP amplification method to determine *Pseudomonas aeruginosa*—a multidrug-resistant infection (Figure 8D) [178]. The outstanding advantage of this sensor is a non-traditional DNA/RNA extraction requirement when Cas12 serves as a collateral cleavage of ssDNA reporter sequences and cognate targets. Additionally, the excess AuNPs-streptavidin complex conjugating in the proposed platform interacted with biotinylated ssDNA reporters and was then captured by a capture probe to spot the sensing performance. This novel approach is inexpensive, ultrasensitive at the very low concentration of 1 CFU/mL, and can especially discriminate a target from both impure and complex non-target samples.

## 9. PADs Engineering-Based Signal Amplification

The last strategy to achieve signal amplification is PAD modification that mainly focuses on design changes to PADs, surface modification of paper or nitrocellulose membrane with the assistance of laser, wax printing, isotachophoresis, or centrifugation.

### 9.1. Design Change of PADs

LFAs commonly consist of multiple components, including a sample pad, a conjugate pad, a membrane, an absorbent pad, and a backing card, making them time-consuming to assemble. By laser cutting, Jiang et al. reported a single-layer LFA constructed from a single piece of cellulose paper to detect the biomarker for malaria infection, *Plasmodium falciparum* histidine-rich protein 2 (*Pf*HRP2), at a 4 ng/mL LOD, visible to the naked eye [183]. Several characteristics of LFA components could impact the performance of LFAs through monitoring the sensing performance of human chorionic gonadotropin. Narrowing-width pads, where the wicking velocity is higher than normal strips, reduce the LOD by half, to 5 mIU/mL, whereas widening the detection pad might achieve color intensity of up to 150%. An optimal distance from the conjugate pad to the test line augments the highest possible color intensity by up to four times [184]. A physical–chemical coupling method using dissoluble saline barriers was developed to enhance LFA sensitivity. After optimizing the design parameters, a 10-fold sensitivity enhancement was achieved, the highest for detection of nucleic acids including HBV, *S. aureus*, and salmonella as model targets [185]. Test-zone pre-enrichment was proposed to improve the LOD of LFA by loading samples before the conjugate pad assembly. After about a 6–8 min pre-enrichment in a running buffer, visual signals were obtained within 20 minutes with a 10-fold to 100-fold improvement for miR-210 mimic and human chorionic gonadotropin protein, compared to conventional LFA [186].

**Figure 9:**
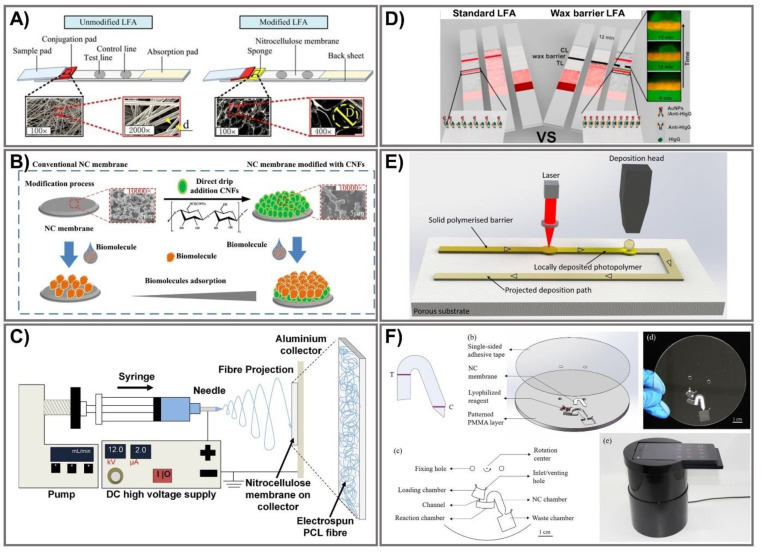
PAD engineering for signal amplification. (**A**) Integration of a sponge shunt into LFA to decrease the fluid flow rate. Adapted with permission from Ref. [174]. (**B**) Modification of an NC membrane with cellulose nanofibers to increase biomolecule adsorption. Adapted with permission from Ref. [190]. (**C**) Direct deposition of polycaprolactone fibers on an NC membrane using electrospin. Adapted with permission from Ref. [191]. (**D**) LFA modified with a wax barrier to decrease the flow rate after the test line. Adapted with permission from Ref. [192]. (**E**) Laser-based direct-write procedure for localized deposition of a photopolymer to create polymerized hydrophobic structures in the porous substrate. Adapted with permission from Ref. [193]. (**F**) Centrifugation-assisted LFA system to control flow rate, including (a) patterned NC membrane, (b) configuration of the centrifugation disc, (c) patterned PMMA layer, (d) assembled LFA disc, and (e) smartphone-based operating devices. Adapted with permission from Ref. [194].

A sponge shunt was integrated into LFA to decrease the fluid flow rate, which could improve sensitivity by signal amplification (Figure 9A). The characteristics of the sponge shunt, including thickness (3 mm), length (7 mm) and hydrophobicity (contact angle of the sponge at 68.5 °C), were optimized to achieve 10-fold signal enhancement compared to the initial LFA. Using AuNPs as colorimetric probes for LFA, this enhancement method based on the addition of a sponge shunt was further confirmed by detecting the Hepatitis B virus (HBV) in clinical serum samples, with LOD of 10^3^ copies/mL for modified LFA, 10 times higher than that of unmodified LFA (10^4^ copies/mL) [174]. Another shunt-integrated LFA was developed to extend the antigen/antibody binding interactions to enhance detection sensitivity to as low as 1 ng/mL of Protein A and 15.5 ng/mL of C-reactive protein, visible with the naked eye (a 1.7-fold enhancement compared to conventional LFA) [187]. The idea of incorporating a piece of a paper-based shunt and a polydimethylsiloxane (PDMS) barrier into the strip was developed to achieve optimum fluidic delays for LFA signal enhancement, resulting in 10-fold signal enhancement over unmodified LFA for detection of HBV [188]. An agarose hybrid as a shunt in LFAs allows sensitive detection of targets with an approximate 10-fold signal improvement [189].

As an alternative to the sponge shunt, Pereira et al. successfully integrated the concentration and detection steps into a single step that occurs entirely within a portable paper-based diagnostic strip. The Triton X-114 micellar aqueous two-phase system (ATPS) was applied to a 3D paper design and effectively reduced the separation time from 8 h to approximately 3 min due to the slower separation speed compared to PEG-phosphate salt ATPS. This 3D design was integrated with LFA to simultaneously concentrate and detect *Plasmodium* lactate dehydrogenase (pLDH) within 20 min with LOD of 1 ng/µL, a 10-fold improvement compared to unmodified LFA [195]. A device integrating a dialysis-based concentration method into LFAs that include a glass fiber-containing PEG buffer and a semi-permeable membrane was also developed to achieve sensitivity improvement in LFAs. PEG acts as a dialysate to selectively absorb small molecules from sample solutions due to a favorable hygroscopic property that enables molecules to diffuse across semi-permeable membranes. The integrated device successfully concentrated and detected Human Immunodeficiency Virus (HIV) nucleic acid and myoglobin with LOD of 0.1 nM and 1.56 ng/mL within 25 min, achieving 10-fold and four-fold signal enhancement, respectively, compared to conventional LFAs [196].

### 9.2. Modification of Nitrocellulose Membrane

An alternative way to manufacture LFAs is to modify the nitrocellulose (NC) membrane to improve the adsorption capacity of biomolecules on PADs such as cellulose nanofibers (CNFs) [190] and chitosan [197]. For instance, CNFs were modified on NC to enhance LFA sensitivity (Figure 9B). CNFs that incorporated NC changed the property of the paper substrate, including a decrease in pore size along with increases in porosity, surface groups, and surface area, and then enhanced the adsorption capacity of biomolecules on the paper substrate. To confirm the enhancing capacity of NCF-based LFAs, both unmodified and modified LFAs were conducted using AuNPs to detect nucleic acid in *S. aureus*. Compared to unmodified LFAs (1 nM), the LOD of CNF-modified LFAs increased by up to 0.05 nM, a 20-fold enhancement [190]. An electrospin method was also explored to adjust the NC membrane in order to reduce the flow rate and increase the interaction rate between the targets and detecting probes [191,198].

Polycaprolactone (PCL) nanofibers were successfully electrospun onto an NC membrane to form a hydrophobic coating, accumulating more immune-complexes (Figure 9C). After a 60 s electrospin duration for PCL-coating the NC, the LFAs achieved a delay of flow as long as 17 ± 1.3 s, increasing the interaction rate of the immune-complex to improve sensitivity of the LFAs to a 0.5 nM LOD for Zika virus cDNA, a 10-fold enhancement compared to an unmodified strip [191]. Instead of laser cutting to reduce the flow rate of LFAs, wax barriers were exploited to control the flow of different solutions in LFAs for sensitivity enhancement. Soluble wax barriers were printed on the NC surface, then melted to penetrate NC pores to create hydrophobic barriers at 1 mm after the test line, temporarily accumulating the target and label nanoparticles on top of the test line (Figure 9D). The wax barriers modulated the internal incubation step of the LFAs to 12 min, promoting immune-complex formation that achieved LOD of 41.19 ng/mL, which was a 51.7-fold sensitivity enhancement, and up to a 96% signal enhancement compared to the unmodified LFA for Human IgG (H-IgG) detection [192]. No full-wax barriers were printed onto the NC membrane, but several wax pillar patterns were printed onto the nitrocellulose membrane in order to produce delays as well as pseudo-turbulence in the microcapillary flow—a sensitivity improvement of almost threefold compared to the sensitivity of a conventional free-barrier LFA [199].

### 9.3. Assistance of Laser Direct Write, Isotachophoresis, or Centrifugation

In order to enhance PAD sensitivity, a laser direct write (LDW) technique is one of the manufacturing approaches used due to intrinsic advantages such as a non-lithographic approach with high flexibility, no special laboratory or material requirements, and the ability to be up-scaled for mass-production of PADs at affordable cost. The implementation of this manufacturing technique involves the deposition of a liquid photopolymer on specific regions of the nitrocellulose membrane, followed by photopolymerization through laser beam irradiation to transform it into impermeable walls, creating fluidic channels as demarcation barriers that transport the liquid samples within the PADs (Figure 9E). The capacity to increase the sensitivity of this LDW method contributes to the slower flow rate and smaller test zone area, which confirmed the improved performance of the LFA strip with C-reactive protein (CRP) for sensitivity enhancement of 62 times and LOD enhancement of 30 times (5 ng/mL compared to the 150 ng/mL of standard LFAs) [193].

Isotachophoresis (ITP), as a powerful electrokinetic pre-concentration and separation technique, was applied to concentrate target analytes into a thin band and transport them to the LFA capture line, resulting in a dramatic increase in the surface reaction rate and equilibrium binding. ITP enhanced sensitivity with a 400-fold LOD improvement for a 90 s assay time, and a 160-fold LOD improvement for a longer 5-min time scale. ITP-enhanced LFA also showed up to a 30% target extraction from 100 μL of the sample, whereas conventional LFA captured less than 1% of the target [200].

Furthermore, centrifugation-assisted LFAs for the detection of PSA have been reported to enhance the sensitivity of LFAs (Figure 9F). A vaulted piece of NC membrane was inserted into a centrifugal disc. Taking advantage of centrifugal force, the sample volume was not limited; the flow rate of the reaction fluid was steady and was adjusted by different rotation speeds with support of the portable operating device constructed to rotate the disc with a stepper motor. By controlling this operating device to achieve the optimal conditions (1500 rpm for a 120 μL sample volume), greater sensitivity was reached at an LOD of 0.067 ng/mL PSA, exhibiting a 6.2-fold improvement in sensitivity compared to conventional LFAs [194]. Therefore, PAD engineering with cost-effectiveness and easy modification holds great promise for biomarker detection with enhanced sensitivity compared to traditional PADs.

## 10. Conclusions

This review provided a comprehensive discussion of state-of-the-art signal amplification strategies used in PAD construction for POC diagnostics. Furthermore, paper microfluidics construction has also revolutionized the existing traditional architectures by generating novel modular devices that can easily be reused and reconfigured. For convenience, various detection techniques employed widely for PAD fabrication were compared in terms of sensing performance (Table 1) to highlight the potential of each method for PAD signal amplification. We focused on PAD modification and signal amplification strategies, including luminescence, colorimetry, and SERS as well as photothermal, photoacoustic, and photoelectrochemical methods, along with nucleic acid-mediated modification. For each signal enhancement method, the basic principles, the classification, and the pros and cons were discussed in detail, and corresponding novel investigations were described. Although the systems proposed in the literature are relatively straightforward, the limits on detection, response time, and sensitivity, as well as their stability, were carefully considered with evaluations of how they applied to real-world applications in analytical devices. Additionally, the integration of nanomaterials into PAD systems was extensively explained with attention to promising approaches designed to facilitate triggering of signal amplification. Based on their exceptional advantages, such as simplicity, disposability, and portability, PAD systems have been applied extensively in various fields of biomedical POC diagnostics, including biomolecules, foodborne bacteria, and especially viral nucleic-acid detection. For instance, during the current COVID-19 pandemic, specific, equipment-free, affordable, and ultrasensitive detection has proved essential for early SARS-CoV-2 diagnoses that can hasten the isolation and treatment of infected people. Despite recognition of current real-time PCR as the best method of SARS-CoV-2 detection, to minimize false negative results and reduce time-consuming public testing, a standard, inexpensive diagnostic tool with wide availability, superb reliability, and rapid response (without equipment) is necessary in order to determine and control viral spread. Collectively, the evolving novel PADs and their corresponding formats serve as flexible and versatile platforms for establishing innovative devices. Moreover, improvements in coming strategies, such as signal amplification modification or nanomaterial incorporation into PAD systems, can offer a synergistic effect, leading to the creation of cost-efficient, single-use, and simple analytical devices. Meeting the crucial point-of-care testing (POCT) criteria (affordable, sensitive, specific, user-friendly, rapid, equipment-free, and easy delivery) will foster the development of more in-home devices with potential clinical applications.

## Figures and Tables

**Table 1:** Performance comparison of different signal amplification strategies for improving sensitivity of PADs.

	Common Probes	Detector	Detection Time	LOD Range	Enhancement
Colorimetric	AuNPs	Camera, naked eye	~10–20 min	0.1–10 ng/mL	~10- to 1000-fold
Luminescent	QDs	Spectrometric detector	~3–120 min	0.01–100 ng/mL	~10- to 50-fold
SERS	AuNPs, AgNPs	Raman spectrophotometer	~30 min	0.1–200 ng/mL	~10- to 100-fold
Photothermal	MnO_2_, AuNPs, GO	IR camera	~15 min	0.4 ng/mL	~10- to 100-fold
Photoacoustic	AuNPs, AgNPs	PA detector	~15 min	0.01 ng/mL	~100-fold
Photoelectrochemical	AuNPs, ZnONPs	Electrochemical device	~30 min	0.6 fM–0.06 nM	~10- to 100-fold
Nucleic acid–mediated	AuNPs, CDs-DNA	Naked eye, photomultiplier tube, camera	~5–30 min	0.15 fM–6.8 nM	~5- to 100-fold
PAD engineering	AuNPs	Camera, naked eye	~15–25 min	0.5–100 ng/mL	~10- to 40-fold

## Data Availability

Not applicable.

## References

[1] Kumar S., Nehra M., Khurana S., Dilbaghi N., Kumar V., Kaushik A., Kim K.-H. (2021). Aspects of Point-of-Care Diagnostics for Personalized Health Wellness. Int. J. Nanomed..

[2] Park H.-D. (2021). Current Status of Clinical Application of Point-of-Care Testing. Arch. Pathol. Lab. Med..

[3] Ferreira C.E.D.S., França C.N., Correr C.J., Zucker M.L., Andriolo A., Scartezini M. (2015). Clinical correlation between a point-of-care testing system and laboratory automation for lipid profile. Clin. Chim. Acta.

[4] Xavier H.T., Ruiz R.M., Júnior L.K., Melone G., Costa W., Fraga R.F., Wajman L., Krakauer M., Scartezini M. (2016). Clinical correlation between the Point-of-care testing method and the traditional clinical laboratory diagnosis in the measure of the lipid profile in patients seen in medical offices. J. Bras. Patol. Med. Lab..

[5] Mahato K., Srivastava A., Chandra P. (2017). Paper based diagnostics for personalized health care: Emerging technologies and commercial aspects. Biosens. Bioelectron..

[6] Hristov D.R., Rodriguez-Quijada C., Gomez-Marquez J., Hamad-Schifferli K. (2019). Designing Paper-Based Immunoassays for Biomedical Applications. Sensors.

[7] Han T., Jin Y., Geng C., Aziz A.U.R., Zhang Y., Deng S., Ren H., Liu B. (2020). Microfluidic Paper-based Analytical Devices in Clinical Applications. Chromatographia.

[8] Ozer T., McMahon C., Henry C.S. (2020). Advances in Paper-Based Analytical Devices. Annu. Rev. Anal. Chem..

[9] Liu L., Yang D., Liu G. (2019). Signal amplification strategies for paper-based analytical devices. Biosens. Bioelectron..

[10] Kim E.B., Cheon S.A., Shim T.S., Kim H.-J., Park T.J. (2020). Reliable naked-eye detection of Mycobacterium tuberculosis antigen 85B using gold and copper nanoshell-enhanced immunoblotting techniques. Sens. Actuators B Chem..

[11] Deng X., Wang C., Gao Y., Li J., Wen W., Zhang X., Wang S. (2018). Applying strand displacement amplification to quantum dots-based fluorescent lateral flow assay strips for HIV-DNA detection. Biosens. Bioelectron..

[12] You M., Lin M., Gong Y., Wang S., Li A., Ji L., Zhao H., Ling K., Wen T., Huang Y. (2017). Household Fluorescent Lateral Flow Strip Platform for Sensitive and Quantitative Prognosis of Heart Failure Using Dual-Color Upconversion Nanoparticles. ACS Nano.

[13] Hwang J., Lee S., Choo J. (2016). Application of a SERS-based lateral flow immunoassay strip for the rapid and sensitive detection of staphylococcal enterotoxin B. Nanoscale.

[14] Tran V., Walkenfort B., König M., Salehi M., Schlücker S. (2019). Rapid, Quantitative, and Ultrasensitive Point-of-Care Testing: A Portable SERS Reader for Lateral Flow Assays in Clinical Chemistry. Angew. Chem. Int. Ed..

[15] Wang Y., Qin Z., Boulware D.R., Pritt B.S., Sloan L.M., González I.J., Bell D., Rees-Channer R.R., Chiodini P., Chan W.C.W. (2016). Thermal Contrast Amplification Reader Yielding 8-Fold Analytical Improvement for Disease Detection with Lateral Flow Assays. Anal. Chem..

[16] Song S., Choi S., Ryu S., Kim S., Kim T., Shin J., Jung H.-I., Joo C. (2018). Highly sensitive paper-based immunoassay using photothermal laser speckle imaging. Biosens. Bioelectron..

[17] Zhao Y., Huang Y., Zhao X., McClelland J.F., Lu M. (2017). Correction: Nanoparticle-based photoacoustic analysis for highly sensitive lateral flow assays. Nanoscale.

[18] Srisomwat C., Yakoh A., Chuaypen N., Tangkijvanich P., Vilaivan T., Chailapakul O. (2021). Amplification-free DNA Sensor for the One-Step Detection of the Hepatitis B Virus Using an Automated Paper-Based Lateral Flow Electrochemical Device. Anal. Chem..

[19] Leichner J., Sarwar M., Nilchian A., Zhu X., Liu H., Shuang S., Li C.-Z. (2017). Electrochemical Lateral Flow Paper Strip for Oxidative-Stress Induced DNA Damage Assessment. Breast Cancer.

[20] Fang Y., Ramasamy R.P. (2015). Current and Prospective Methods for Plant Disease Detection. Biosensors.

[21] Yamada K., Shibata H., Suzuki K., Citterio D. (2017). Toward practical application of paper-based microfluidics for medical diagnostics: State-of-the-art and challenges. Lab Chip.

[22] Li F., Wang X., Liu J., Hu Y., He J. (2019). Double-layered microfluidic paper-based device with multiple colorimetric indicators for multiplexed detection of biomolecules. Sens. Actuators B Chem..

[23] Ali M.M., Wolfe M., Tram K., Gu J., Filipe C.D.M., Li Y., Brennan J.D. (2019). A DNAzyme-Based Colorimetric Paper Sensor for Helicobacter pylori. Angew. Chem. Int. Ed..

[24] Abarghoei S., Fakhri N., Borghei Y.S., Hosseini M., Ganjali M.R. (2019). A colorimetric paper sensor for citrate as biomarker for early stage detection of prostate cancer based on peroxidase-like activity of cysteine-capped gold nanoclusters. Spectrochim. Acta Part A Mol. Biomol. Spectrosc..

[25] Alahmad W., Tungkijanansin N., Kaneta T., Varanusupakul P. (2018). A colorimetric paper-based analytical device coupled with hollow fiber membrane liquid phase microextraction (HF-LPME) for highly sensitive detection of hexavalent chromium in water samples. Talanta.

[26] Devadhasan J.P., Kim J. (2018). A chemically functionalized paper-based microfluidic platform for multiplex heavy metal detection. Sens. Actuators B Chem..

[27] Li F., Hu Y., Li Z., Liu J., Guo L., He J. (2019). Three-dimensional microfluidic paper-based device for multiplexed colorimetric detection of six metal ions combined with use of a smartphone. Anal. Bioanal. Chem..

[28] Nouanthavong S., Nacapricha D., Henry C.S., Sameenoi Y. (2016). Pesticide analysis using nanoceria-coated paper-based devices as a detection platform. Analyst.

[29] Bordbar M.M., Nguyen T.A., Arduini F., Bagheri H. (2020). A paper-based colorimetric sensor array for discrimination and simultaneous determination of organophosphate and carbamate pesticides in tap water, apple juice, and rice. Microchim. Acta.

[30] Fu Q., Zhang C., Xie J., Li Z., Qu L., Cai X., Ouyang H., Song Y., Du D., Lin Y. (2019). Ambient light sensor based colorimetric dipstick reader for rapid monitoring organophosphate pesticides on a smart phone. Anal. Chim. Acta.

[31] Sheini A. (2020). Colorimetric aggregation assay based on array of gold and silver nanoparticles for simultaneous analysis of aflatoxins, ochratoxin and zearalenone by using chemometric analysis and paper based analytical devices. Microchim. Acta.

[32] Najafzadeh F., Ghasemi F., Hormozi-Nezhad M.R. (2018). Anti-aggregation of gold nanoparticles for metal ion discrimination: A promising strategy to design colorimetric sensor arrays. Sens. Actuators B Chem..

[33] Pinyorospathum C., Rattanarat P., Chaiyo S., Siangproh W., Chailapakul O. (2019). Colorimetric sensor for determination of phosphate ions using anti-aggregation of 2-mercaptoethanesulfonate-modified silver nanoplates and europium ions. Sens. Actuators B Chem..

[34] Kong Q., Wang Y., Zhang L., Ge S., Yu J. (2017). A novel microfluidic paper-based colorimetric sensor based on molecularly imprinted polymer membranes for highly selective and sensitive detection of bisphenol A. Sens. Actuators B Chem..

[35] Sharifi H., Tashkhourian J., Hemmateenejad B. (2020). A 3D origami paper-based analytical device combined with PVC membrane for colorimetric assay of heavy metal ions: Application to determination of Cu(II) in water samples. Anal. Chim. Acta.

[36] Xu X., Wang L., Zou X., Wu S., Pan J., Li X., Niu X. (2019). Highly sensitive colorimetric detection of arsenite based on reassembly-induced oxidase-mimicking activity inhibition of dithiothreitol-capped Pd nanozyme. Sens. Actuators B Chem..

[37] Sengupta P., Pramanik K., Datta P., Sarkar P. (2020). Chemically modified carbon nitride-chitin-acetic acid hybrid as a metal-free bifunctional nanozyme cascade of glucose oxidase-peroxidase for “click off” colorimetric detection of peroxide and glucose. Biosens. Bioelectron..

[38] Weerathunge P., Ramanathan R., Torok V.A., Hodgson K., Xu Y., Goodacre R., Behera B.K., Bansal V. (2019). Ultrasensitive Colorimetric Detection of Murine Norovirus Using NanoZyme Aptasensor. Anal. Chem..

[39] Ko E., Tran V.-K., Son S.E., Hur W., Choi H., Seong G.H. (2019). Characterization of Au@PtNP/GO nanozyme and its application to electrochemical microfluidic devices for quantification of hydrogen peroxide. Sens. Actuators B Chem..

[40] Choleva T.G., Kappi F.A., Giokas D.L., Vlessidis A.G. (2015). Paper-based assay of antioxidant activity using analyte-mediated on-paper nucleation of gold nanoparticles as colorimetric probes. Anal. Chim. Acta.

[41] Gupta R., Kumar A., Kumar S., Pinnaka A.K., Singhal N.K. (2021). Naked eye colorimetric detection of Escherichia coli using aptamer conjugated graphene oxide enclosed Gold nanoparticles. Sens. Actuators B Chem..

[42] Kaushal S., Pinnaka A.K., Soni S., Singhal N.K. (2021). Antibody assisted graphene oxide coated gold nanoparticles for rapid bacterial detection and near infrared light enhanced antibacterial activity. Sens. Actuators B Chem..

[43] Rodríguez M.O., Covián L.B., García A.C., Blanco-López M.C. (2016). Silver and gold enhancement methods for lateral flow immunoassays. Talanta.

[44] Zhu X., Huang J., Liu J., Zhang H., Jiang J., Yu R. (2017). A dual enzyme–inorganic hybrid nanoflower incorporated microfluidic paper-based analytic device (μPAD) biosensor for sensitive visualized detection of glucose. Nanoscale.

[45] Panferov V.G., Safenkova I.V., Zherdev A.V., Dzantiev B.B. (2018). Post-assay growth of gold nanoparticles as a tool for highly sensitive lateral flow immunoassay. Application to the detection of potato virus X. Microchim. Acta.

[46] Chun P., Metzler J.B. (2008). Colloidal Gold and Other Labels for Lateral Flow Immunoassays. Lateral Flow Immunoassay.

[47] Kim J.-H., Park J.-E., Lin M., Kim S., Kim G.-H., Park S., Ko G., Nam J.-M. (2017). Sensitive, Quantitative Naked-Eye Biodetection with Polyhedral Cu Nanoshells. Adv. Mater..

[48] Phan L.M.T., Rafique R., Baek S.H., Nguyen T.P., Park K.Y., Kim E.B., Gil Kim J., Park J.P., Kailasa S.K., Kim H.-J. (2018). Gold-copper nanoshell dot-blot immunoassay for naked-eye sensitive detection of tuberculosis specific CFP-10 antigen. Biosens. Bioelectron..

[49] Mazur F., Tran H., Kuchel R.P., Chandrawati R. (2020). Rapid Detection of Listeriolysin O Toxin Based on a Nanoscale Liposome–Gold Nanoparticle Platform. ACS Appl. Nano Mater..

[50] You S.-M., Jeong K.-B., Luo K., Park J.-S., Park J.-W., Kim Y.-R. (2021). Paper-based colorimetric detection of pathogenic bacteria in food through magnetic separation and enzyme-mediated signal amplification on paper disc. Anal. Chim. Acta.

[51] Pang H.-H., Ke Y.-C., Li N.-S., Chen Y.-T., Huang C.-Y., Wei K.-C., Yang H.-W. (2020). A new lateral flow plasmonic biosensor based on gold-viral biomineralized nanozyme for on-site intracellular glutathione detection to evaluate drug-resistance level. Biosens. Bioelectron..

[52] Wu S., Li D., Wang J., Zhao Y., Dong S., Wang X. (2017). Gold nanoparticles dissolution based colorimetric method for highly sensitive detection of organophosphate pesticides. Sens. Actuators B Chem..

[53] Memon S.S., Nafady A., Solangi A.R., Al-Enizi A.M., Sirajuddin, Shah M.R., Sherazi S.T., Memon S., Arain M., Abro M.I. (2018). Sensitive and selective aggregation based colorimetric sensing of Fe^3+^ via interaction with acetyl salicylic acid derived gold nanoparticles. Sens. Actuators B Chem..

[54] Zhang Y., Wang H., Xiao S., Wang X., Xu P. (2020). A triple functional sensing chip for rapid detection of pathogenic Listeria monocytogenes. bioRxiv.

[55] Díaz-Amaya S., Zhao M., Allebach J.P., Chiu G.T.-C., Stanciu L.A. (2020). Ionic Strength Influences on Biofunctional Au-Decorated Microparticles for Enhanced Performance in Multiplexed Colorimetric Sensors. ACS Appl. Mater. Interfaces.

[56] Basiri S., Mehdinia A., Jabbari A. (2018). Green synthesis of reduced graphene oxide-Ag nanoparticles as a dual-responsive colorimetric platform for detection of dopamine and Cu2+. Sens. Actuators B Chem..

[57] Böhm A., Trosien S., Avrutina O., Kolmar H., Biesalski M. (2018). Covalent Attachment of Enzymes to Paper Fibers for Paper-Based Analytical Devices. Front. Chem..

[58] Zhang Y., Pan D., Zhou Q., Zhao J., Pan N., Zhang Y., Wang L.-X., Shen Y. (2018). An enzyme cascade-based electrochemical immunoassay using a polydopamine–carbon nanotube nanocomposite for signal amplification. J. Mater. Chem. B.

[59] Jin R., Kong D., Zhao X., Li H., Yan X., Liu F., Sun P., Du D., Lin Y., Lu G. (2019). Tandem catalysis driven by enzymes directed hybrid nanoflowers for on-site ultrasensitive detection of organophosphorus pesticide. Biosens. Bioelectron..

[60] Koua X., Tonga L., Shena Y., Zhub W., Yina L., Huangb S., Zhua F., Chena G., Ouyanga G. (2020). Smartphone-assisted robust enzymes@MOFs-based paper biosensor for point-of-care detection. Biosens. Bioelectron..

[61] Kizling M., Dzwonek M., Nowak A., Tymecki Ł., Stolarczyk K., Więckowska A., Bilewicz R. (2020). Multi-Substrate Biofuel Cell Utilizing Glucose, Fructose and Sucrose as the Anode Fuels. Nanomaterials.

[62] Luckham R.E., Brennan J.D. (2010). Bioactive paper dipstick sensors for acetylcholinesterase inhibitors based on sol–gel/enzyme/gold nanoparticle composites. Analyst.

[63] Xu Y., Li F., Yang K., Qiao Y., Yan Y., Yan J. (2019). A facile and robust non-natural three enzyme biocatalytic cascade based on Escherichia coli surface assembly for fatty alcohol production. Energy Convers. Manag..

[64] Zinna J., Lockwood T.-L.E., Lieberman M. (2020). Enzyme-based paper test for detection of lactose in illicit drugs. Anal. Methods.

[65] Zhao Y., Zeng D., Yan C., Chen W., Ren J., Jiang Y., Jiang L., Xue F., Ji D., Tang F. (2020). Rapid and accurate detection of Escherichia coli O157:H7 in beef using microfluidic wax-printed paper-based ELISA. Analyst.

[66] Swain K.K., Bhand S. (2021). A colorimetric paper-based ATONP-ALP nanobiosensor for selective detection of Cd2+ ions in clams and mussels. Anal. Bioanal. Chem..

[67] Li W., Lu S., Bao S., Shi Z., Lu Z., Li C., Yu L. (2018). Efficient in situ growth of enzyme-inorganic hybrids on paper strips for the visual detection of glucose. Biosens. Bioelectron..

[68] Wang H., Wan K., Shi X. (2019). Recent Advances in Nanozyme Research. Adv. Mater..

[69] Xu B., Cui Y., Wang W., Li S., Lyu C., Wang S., Bao W., Wang H., Qin M., Liu Z. (2020). Immunomodulation-Enhanced Nanozyme-Based Tumor Catalytic Therapy. Adv. Mater..

[70] Tomei M.R., Cinti S., Interino N., Manovella V., Moscone D., Arduini F. (2019). Paper-based electroanalytical strip for user-friendly blood glutathione detection. Sens. Actuators B Chem..

[71] Huang L., Sun D.-W., Pu H., Wei Q., Luo L., Wang J. (2019). A colorimetric paper sensor based on the domino reaction of acetylcholinesterase and degradable γ-MnOOH nanozyme for sensitive detection of organophosphorus pesticides. Sens. Actuators B Chem..

[72] Han T., Wang G. (2019). Peroxidase-like activity of acetylcholine-based colorimetric detection of acetylcholinesterase activity and an organophosphorus inhibitor. J. Mater. Chem. B.

[73] Liu F., Zhang C. (2015). A novel paper-based microfluidic enhanced chemiluminescence biosensor for facile, reliable and highly-sensitive gene detection of Listeria monocytogenes. Sens. Actuators B Chem..

[74] Sutariya P.G., Soni H., Gandhi S.A., Pandya A. (2019). Novel luminescent paper based calix[4]arene chelation enhanced fluorescence- photoinduced electron transfer probe for Mn^2+^, Cr^3+^ and F^−^. J. Lumin..

[75] Wang Q., Wei H., Zhang Z., Wang E., Dong S. (2018). Nanozyme: An emerging alternative to natural enzyme for biosensing and immunoassay. TrAC Trends Anal. Chem..

[76] She P., Ma Y., Qin Y., Xie M., Li F., Liu S., Huang W., Zhao Q. (2019). Dynamic Luminescence Manipulation for Rewritable and Multi-level Security Printing. Matter.

[77] Liu C., Zhang R., Zhang W., Liu J., Wang Y.-L., Du Z., Song B., Xu Z.P., Yuan J. (2019). “Dual-Key-and-Lock” Ruthenium Complex Probe for Lysosomal Formaldehyde in Cancer Cells and Tumors. J. Am. Chem. Soc..

[78] Taprab N., Sameenoi Y. (2019). Rapid screening of formaldehyde in food using paper-based titration. Anal. Chim. Acta.

[79] Sun X., Li B., Tian C., Yu F., Zhou N., Zhan Y., Chen L. (2018). Rotational paper-based electrochemiluminescence immunodevices for sensitive and multiplexed detection of cancer biomarkers. Anal. Chim. Acta.

[80] Chen Y., Guo X., Liu W., Zhang L. (2019). Paper-based fluorometric immunodevice with quantum-dot labeled antibodies for simultaneous detection of carcinoembryonic antigen and prostate specific antigen. Microchim. Acta.

[81] Sun H., Li W., Dong Z.-Z., Hu C., Leung C.-H., Ma D.-L., Ren K. (2018). A suspending-droplet mode paper-based microfluidic platform for low-cost, rapid, and convenient detection of lead(II) ions in liquid solution. Biosens. Bioelectron..

[82] Othong J., Boonmak J., Kielar F., Youngme S. (2020). Dual Function Based on Switchable Colorimetric Luminescence for Water and Temperature Sensing in Two-Dimensional Metal–Organic Framework Nanosheets. ACS Appl. Mater. Interfaces.

[83] Dunning S.G., Nuñez A.J., Moore M.D., Steiner A., Lynch V.M., Sessler J.L., Holliday B.J., Humphrey S.M. (2017). A Sensor for Trace H_2_O Detection in D_2_O. Chem.

[84] Lin L.-K., Stanciu L.A. (2018). Bisphenol A detection using gold nanostars in a SERS improved lateral flow immunochromatographic assay. Sens. Actuators B Chem..

[85] Lin L.-K., Uzunoglu A., Stanciu L.A. (2018). Aminolated and Thiolated PEG-Covered Gold Nanoparticles with High Stability and Antiaggregation for Lateral Flow Detection of Bisphenol A. Small.

[86] Zhao P., Liu H., Zhang L., Zhu P., Ge S., Yu J. (2020). Paper-Based SERS Sensing Platform Based on 3D Silver Dendrites and Molecularly Imprinted Identifier Sandwich Hybrid for Neonicotinoid Quantification. ACS Appl. Mater. Interfaces.

[87] Chen S., Gao J., Chang J., Zhang Y., Feng L. (2019). Organic-inorganic manganese (II) halide hybrids based paper sensor for the fluorometric determination of pesticide ferbam. Sens. Actuators B Chem..

[88] Park M., Hwang C.S.H., Jeong K.-H. (2018). Nanoplasmonic Alloy of Au/Ag Nanocomposites on Paper Substrate for Biosensing Applications. ACS Appl. Mater. Interfaces.

[89] Li W., Zhang X., Miao C., Li R., Ji Y. (2020). Fluorescent paper–based sensor based on carbon dots for detection of folic acid. Anal. Bioanal. Chem..

[90] Huang D., Zhuang Z., Wang Z., Li S., Zhong H., Liu Z., Guo Z., Zhang W. (2019). Black phosphorus-Au filter paper-based three-dimensional SERS substrate for rapid detection of foodborne bacteria. Appl. Surf. Sci..

[91] Weng G., Yang Y., Zhao J., Li J., Zhu J., Zhao J. (2020). Improving the SERS enhancement and reproducibility of inkjet-printed Au NP paper substrates by second growth of Ag nanoparticles. Mater. Chem. Phys..

[92] Liu H., Guo Y., Wang Y., Zhang H., Ma X., Wen S., Jin J., Song W., Zhao B., Ozaki Y. (2021). A nanozyme-based enhanced system for total removal of organic mercury and SERS sensing. J. Hazard. Mater..

[93] Ponram M., Balijapalli U., Sambath B., Iyer S.K., Venkatachalapathy B., Cingaram R., Sundaramurthy K.N. (2018). Development of paper-based chemosensor for the detection of mercury ions using mono- and tetra-sulfur bearing phenanthridines. New J. Chem..

[94] Hwang S., Nam J., Jung S., Song J., Doh H., Kim S. (2014). Gold nanoparticle-mediated photothermal therapy: Current status and future perspective. Nanomedicine.

[95] Han B., Zhang Y.-L., Chen Q.-D., Sun H.-B. (2018). Carbon-Based Photothermal Actuators. Adv. Funct. Mater..

[96] Tao W., Ji X., Xu X., Islam M.A., Li Z., Chen S., Saw P.E., Zhang H., Bharwani Z., Guo Z. (2017). Antimonene Quantum Dots: Synthesis and Application as Near-Infrared Photothermal Agents for Effective Cancer Therapy. Angew. Chem..

[97] Kim M., Lee J., Nam J. (2019). Plasmonic Photothermal Nanoparticles for Biomedical Applications. Adv. Sci..

[98] Riley R.S., Day E.S. (2017). Gold nanoparticle-mediated photothermal therapy: Applications and opportunities for multimodal cancer treatment. Wiley Interdiscip. Rev. Nanomed. Nanobiotechnol..

[99] Yang W., Liang H., Ma S., Wang D., Huang J. (2019). Gold nanoparticle based photothermal therapy: Development and application for effective cancer treatment. Sustain. Mater. Technol..

[100] Su L., Chen Y., Wang L., Zhang H., Sun J., Wang J., Zhang D. (2021). Dual-signal based immunoassay for colorimetric and photothermal detection of furazolidone. Sens. Actuators B Chem..

[101] Liu S., Dou L., Yao X., Zhang W., Zhao B., Wang Z., Ji Y., Sun J., Xu B., Zhang D. (2020). Polydopamine nanospheres as high-affinity signal tag towards lateral flow immunoassay for sensitive furazolidone detection. Food Chem..

[102] Fu G., Zhu Y., Wang W., Zhou M., Li X. (2019). Spatiotemporally Controlled Multiplexed Photothermal Microfluidic Pumping under Monitoring of On-Chip Thermal Imaging. ACS Sens..

[103] Fu G., Li X., Wang W., Hou R. (2020). Multiplexed tri-mode visual outputs of immunoassay signals on a clip-magazine-assembled photothermal biosensing disk. Biosens. Bioelectron..

[104] Shen C., Zhu Y., Xiao X., Xu X., Chen X., Xu G. (2020). Economical Salt-Resistant Superhydrophobic Photothermal Membrane for Highly Efficient and Stable Solar Desalination. ACS Appl. Mater. Interfaces.

[105] Fu G., Zhu Y., Xu K., Wang W., Hou R., Li X. (2019). Photothermal Microfluidic Sensing Platform Using Near-Infrared Laser-Driven Multiplexed Dual-Mode Visual Quantitative Readout. Anal. Chem..

[106] Zhang L., Sun L., Hou M., Xu Z., Kang Y., Xue P. (2018). A paper-based photothermal array using Parafilm to analyze hyperthermia response of tumour cells under local gradient temperature. Biomed. Microdevices.

[107] Nilghaz A., Wicaksono D.H.B., Gustiono D., Majid F.A.A., Supriyanto E., Kadir M.R.A. (2011). Flexible microfluidic cloth-based analytical devices using a low-cost waxpatterning technique. Lab Chip.

[108] Fu G., Sanjay S.T., Li X. (2016). Cost-effective and sensitive colorimetric immunosensing using an iron oxide-to-Prussian blue nanoparticle conversion strategy. Analyst.

[109] Fu G., Sanjay S.T., Zhou W., Brekken R.A., Kirken R.A., Li X. (2018). Exploration of Nanoparticle-Mediated Photothermal Effect of TMB-H2O2 Colorimetric System and Its Application in a Visual Quantitative Photothermal Immunoassay. Anal. Chem..

[110] Li X., Xu W., Tang M., Zhou L., Zhu B., Zhu S., Zhu J. (2016). Graphene oxide-based efficient and scalable solar desalination under one sun with a confined 2D water path. Proc. Natl. Acad. Sci. USA.

[111] Zhao J., Yang Y., Yang C., Tian Y., Han Y., Liu J., Yin X., Que W. (2018). A hydrophobic surface enabled salt-blocking 2D Ti3C2MXene membrane for efficient and stable solar desalination. J. Mater. Chem. A.

[112] Zhao Y., Huang Y., Zhao X.-W., McClelland J.F., Lu M. (2016). Nanoparticle-based photoacoustic analysis for highly sensitive lateral flow assays. Nanoscale.

[113] Jeevarathinam A.S., Pai N., Huang K., Hariri A., Wang J., Bai Y., Wang L., Hancock T., Keys S., Penny W. (2019). A cellulose-based photoacoustic sensor to measure heparin concentration and activity in human blood samples. Biosens. Bioelectron..

[114] Zhang Y.-J., Guo L., Chen S., Yu Y.-L., Wang J.-H. (2020). A portable photoacoustic device for facile and sensitive detection of serum alkaline phosphatase activity. Anal. Chim. Acta.

[115] Gray J.P., Dana N., Dextraze K.L., Maier F., Emelianov S., Bouchard R.R. (2015). Multi-Wavelength Photoacoustic Visualization of High Intensity Focused Ultrasound Lesions. Ultrason. Imaging.

[116] Ye H., Liu Y., Zhan L., Liu Y., Qin Z. (2020). Signal amplification and quantification on lateral flow assays by laser excitation of plasmonic nanomaterials. Theranostics.

[117] Gao C., Yu H., Zhang L., Zhao Y., Xie J., Li C., Cui K., Yu J. (2020). Ultrasensitive Paper-Based Photoelectrochemical Sensing Platform Enabled by the Polar Charge Carriers-Created Electric Field. Anal. Chem..

[118] Fu L.-M., Wang Y.-N. (2018). Detection methods and applications of microfluidic paper-based analytical devices. TrAC Trends Anal. Chem..

[119] Gao C., Xue J., Zhang L., Cui K., Li H., Yu J. (2018). Paper-Based Origami Photoelectrochemical Sensing Platform with TiO_2_/Bi4NbO8Cl/Co-Pi Cascade Structure Enabling of Bidirectional Modulation of Charge Carrier Separation. Anal. Chem..

[120] Kong Q., Wang Y., Zhang L., Xu C., Yu J. (2018). Highly sensitive microfluidic paper-based photoelectrochemical sensing platform based on reversible photo-oxidation products and morphology-preferable multi-plate ZnO nanoflowers. Biosens. Bioelectron..

[121] Svitkova V., Palchetti I. (2020). Functional polymers in photoelectrochemical biosensing. Bioelectrochemistry.

[122] Zhao C.-Q., Ding S.-N. (2019). Perspective on signal amplification strategies and sensing protocols in photoelectrochemical immunoassay. Coord. Chem. Rev..

[123] Yang H., Zhang Y., Zhang L., Cui K., Ge S., Huang J., Yu J. (2018). Stackable Lab-on-Paper Device with All-in-One Au Electrode for High-Efficiency Photoelectrochemical Cyto-Sensing. Anal. Chem..

[124] Yang H., Hu M., Li Z., Zhao P., Xie L., Song X., Yu J. (2019). Donor/Acceptor-Induced Ratiometric Photoelectrochemical Paper Analytical Device with a Hollow Double-Hydrophilic-Walls Channel for microRNA Quantification. Anal. Chem..

[125] Medetalibeyoglua H., Kotan G., Atarc N., Yola M.L. (2020). A novel sandwich-type SERS immunosensor for selective and sensitive carcinoembryonic antigen (CEA) detection. Anal. Chim. Acta.

[126] Xue J., Zhang L., Gao C., Zhu P., Yu J. (2019). Microfluidic paper-based photoelectrochemical sensing platform with electron-transfer tunneling distance regulation strategy for thrombin detection. Biosens. Bioelectron..

[127] Gao C., Xue J., Zhang L., Zhao P., Cui K., Ge S., Yu J. (2019). Paper based modification-free photoelectrochemical sensing platform with single-crystalline aloe like TiO_2_ as electron transporting material for cTnI detection. Biosens. Bioelectron..

[128] Li L., Zhang Y., Yan Z., Chen M., Zhang L., Zhao P., Yu J. (2020). Ultrasensitive Photoelectrochemical Detection of MicroRNA on Paper by Combining a Cascade Nanozyme-Engineered Biocatalytic Precipitation Reaction and Target-Triggerable DNA Motor. ACS Sens..

[129] Wang S., Zhao J., Zhang Y., Yan M., Zhang L., Ge S., Yu J. (2019). Photoelectrochemical biosensor of HIV-1 based on cascaded photoactive materials and triple-helix molecular switch. Biosens. Bioelectron..

[130] Kong Q., Cui K., Zhang L., Wang Y., Sun J., Ge S., Zhang Y., Yu J. (2018). “On–Off–On” Photoelectrochemical/Visual Lab-on-Paper Sensing via Signal Amplification of CdS Quantum Dots@Leaf-Shape ZnO and Quenching of Au-Modified Prism-Anchored Octahedral CeO2 Nanoparticles. Anal. Chem..

[131] Shi H., Ge S., Wang Y., Gao C., Yu J. (2019). Wide-Spectrum-Responsive Paper-Supported Photoelectrochemical Sensing Platform Based on Black Phosphorus-Sensitized TiO_2_. ACS Appl. Mater. Interfaces.

[132] Zhang L., Kong Q., Li L., Wang Y., Ge S., Yu J. (2021). Direct-readout photoelectrochemical lab-on-paper biosensing platform based on coupled electricity generating system and paper supercapacitors. Talanta.

[133] Jiang Y., Yang Y., Zheng X., Yi Y., Chen X., Li Y., Sun D., Zhang L. (2020). Multifunctional load-bearing hybrid hydrogel with combined drug release and photothermal conversion functions. NPG Asia Mater..

[134] Sun J., Li L., Kong Q., Zhang Y., Zhao P., Ge S., Cui K., Yu J. (2019). Mimic peroxidase-transfer enhancement of photoelectrochemical aptasensing via CuO nanoflowers functionalized lab-on-paper device with a controllable fluid separator. Biosens. Bioelectron..

[135] Hua M., Yanga H., Lia Z., Zhangb L., Zhua P., Yana M., Yua J. (2020). Signal-switchable lab-on-paper photoelectrochemical aptasensing system integrated triple-helix molecular switch with charge separation and recombination regime of type-II CdTe@CdSe core-shell quantum dots. Biosens. Bioelectron..

[136] Yang R., Li F., Zhang W., Shen W., Yang D., Bian Z., Cui H. (2019). Chemiluminescence Immunoassays for Simultaneous Detection of Three Heart Disease Biomarkers Using Magnetic Carbon Composites and Three-Dimensional Microfluidic Paper-Based Device. Anal. Chem..

[137] Ge S., Liang L., Lan F., Zhang Y., Wang Y., Yan M., Yu J. (2016). Photoelectrochemical immunoassay based on chemiluminescence as internal excited light source. Sens. Actuators B Chem..

[138] Wang Y., Liu H., Wang P., Yu J., Ge S., Yan M. (2015). Chemiluminescence excited photoelectrochemical competitive immunosensing lab-on-paper device using an integrated paper supercapacitor for signal amplication. Sens. Actuators B Chem..

[139] Sun G., Zhang Y., Kong Q., Ma C., Yu J., Ge S., Yan M., Song X. (2014). Chemiluminescence excited paper-based photoelectrochemical competitive immunosensing based on porous ZnO spheres and CdS nanorods. J. Mater. Chem. B.

[140] Ge S., Lan F., Liang L., Ren N., Li L., Liu H., Yan M., Yu J. (2017). Ultrasensitive Photoelectrochemical Biosensing of Cell Surface N-Glycan Expression Based on the Enhancement of Nanogold-Assembled Mesoporous Silica Amplified by Graphene Quantum Dots and Hybridization Chain Reaction. ACS Appl. Mater. Interfaces.

[141] Xu X., Wang J., Wang Y., Zhao L., Li Y., Liu C. (2018). Formation of graphene oxide-hybridized nanogels for combinative anticancer therapy. Nanomed. Nanotechnol. Biol. Med..

[142] Lan F., Liang L., Zhang Y., Li L., Ren N., Yan M., Ge S., Yu J. (2017). Internal Light Source-Driven Photoelectrochemical 3D-rGO/Cellulose Device Based on Cascade DNA Amplification Strategy Integrating Target Analog Chain and DNA Mimic Enzyme. ACS Appl. Mater. Interfaces.

[143] Li Z., Yang H., Hu M., Zhang L., Ge S., Cui K., Yu J. (2020). Cathode Photoelectrochemical Paper Device for microRNA Detection Based on Cascaded Photoactive Structures and Hemin/Pt Nanoparticle-Decorated DNA Dendrimers. ACS Appl. Mater. Interfaces.

[144] Li L., Wang T., Zhang Y., Xu C., Zhang L., Cheng X., Liu H., Chen X., Yu J. (2018). Editable TiO_2_ Nanomaterial-Modified Paper in Situ for Highly Efficient Detection of Carcinoembryonic Antigen by Photoelectrochemical Method. ACS Appl. Mater. Interfaces.

[145] Zeng R., Luo Z., Zhang L., Tang D. (2018). Platinum Nanozyme-Catalyzed Gas Generation for Pressure-Based Bioassay Using Polyaniline Nanowires-Functionalized Graphene Oxide Framework. Anal. Chem..

[146] Chen W., Fang X., Li H., Cao H., Kong J. (2017). DNA-mediated inhibition of peroxidase-like activities on platinum nanoparticles for simple and rapid colorimetric detection of nucleic acids. Biosens. Bioelectron..

[147] Liu M., Wang J., Chang Y., Zhang Q., Chang D., Hui C.Y., Brennan J.D., Li Y. (2020). In Vitro Selection of a DNA Aptamer Targeting Degraded Protein Fragments for Biosensing. Angew. Chem. Int. Ed..

[148] Wang X., Chen X., Chu C., Deng Y., Yang M., Ji Z., Xu F., Huo D., Luo Y., Hou C. (2020). Four-stage signal amplification for trace ATP detection using allosteric probe-conjugated strand displacement and CRISPR/Cpf1 trans-cleavage (ASD-Cpf1). Sens. Actuators B Chem..

[149] Tian T., Bi Y., Xu X., Zhu Z., Yang C.J. (2018). Integrated paper-based microfluidic devices for point-of-care testing. Anal. Methods.

[150] Magro L., Escadafal C., Garneret P., Jacquelin B., Kwasiborski A., Manuguerra J.-C., Monti F., Sakuntabhai A., Vanhomwegen J., Lafaye P. (2017). Paper microfluidics for nucleic acid amplification testing (NAAT) of infectious diseases. Lab Chip.

[151] Wu L., Ma C., Zheng X., Liu H., Yu J. (2015). Paper-based electrochemiluminescence origami device for protein detection using assembled cascade DNA–carbon dots nanotags based on rolling circle amplification. Biosens. Bioelectron..

[152] Bialy R.M., Ali M.M., Li Y., Brennan J.D. (2020). Protein-Mediated Suppression of Rolling Circle Amplification for Biosensing with an Aptamer-Containing DNA Primer. Chem. Eur. J..

[153] Sun Y., Chang Y., Zhang Q., Liu M. (2019). An Origami Paper-Based Device Printed with DNAzyme-Containing DNA Superstructures for Escherichia coli Detection. Micromachines.

[154] Li X., He X., Zhang Q., Chang Y., Liu M. (2019). Graphene oxide-circular aptamer based colorimetric protein detection on bioactive paper. Anal. Methods.

[155] Gyanchandani R., Kvam E., Heller R., Finehout E., Smith N., Kota K., Nelson J.R., Griffin W., Puhalla S., Brufsky A.M. (2018). Whole genome amplification of cell-free DNA enables detection of circulating tumor DNA mutations from fingerstick capillary blood. Sci. Rep..

[156] Phillips E.A., Moehling T.J., Bhadra S., Ellington A.D., Linnes J.C. (2018). Strand Displacement Probes Combined with Isothermal Nucleic Acid Amplification for Instrument-Free Detection from Complex Samples. Anal. Chem..

[157] Kaarj K., Akarapipad P., Yoon J.-Y. (2018). Simpler, Faster, and Sensitive Zika Virus Assay Using Smartphone Detection of Loop-mediated Isothermal Amplification on Paper Microfluidic Chips. Sci. Rep..

[158] Seok Y., Joung H.-A., Byun J.-Y., Jeon H.-S., Shin S.J., Kim S., Shin Y.-B., Han H.S., Kim M.-G. (2017). A Paper-Based Device for Performing Loop-Mediated Isothermal Amplification with Real-Time Simultaneous Detection of Multiple DNA Targets. Theranostics.

[159] Choi J.R., Hu J., Gong Y., Feng S., Abas W.A.B.W., Pingguan-Murphy B., Xu F. (2016). An integrated lateral flow assay for effective DNA amplification and detection at the point of care. Analyst.

[160] Hui J., Gu Y., Zhu Y., Chen Y., Guo S.-J., Tao S.-C., Zhang Y., Liu P. (2018). Multiplex sample-to-answer detection of bacteria using a pipette-actuated capillary array comb with integrated DNA extraction, isothermal amplification, and smartphone detection. Lab Chip.

[161] Naik P., Jaitpal S., Shetty P., Paul D. (2019). An integrated one-step assay combining thermal lysis and loop-mediated isothermal DNA amplification (LAMP) in 30 min from E. coli and M. smegmatis cells on a paper substrate. Sens. Actuators B Chem..

[162] Trinh T.N.D., Lee N.Y. (2019). A foldable isothermal amplification microdevice for fuchsin-based colorimetric detection of multiple foodborne pathogens. Lab Chip.

[163] Choi J.R., Hu J., Tang R., Gong Y., Feng S., Ren H., Wen T., Li X., Abas W.A.B.W., Pingguan-Murphy B. (2015). An integrated paper-based sample-to-answer biosensor for nucleic acid testing at the point of care. Lab Chip.

[164] Safavieh M., Kaul V., Khetani S., Singh A., Dhingra K., Kanakasabapathy M.K., Draz M.S., Memic A., Kuritzkes D.R., Shafiee H. (2016). Paper microchip with a graphene-modified silver nano-composite electrode for electrical sensing of microbial pathogens. Nanoscale.

[165] Yang Z., Xu G., Reboud J., Ali S.A., Kaur G., McGiven J., Boby N., Gupta P.K., Chaudhuri P., Cooper J.M. (2018). Rapid Veterinary Diagnosis of Bovine Reproductive Infectious Diseases from Semen Using Paper-Origami DNA Microfluidics. ACS Sens..

[166] Saengsawang N., Ruang-Areerate T., Kesakomol P., Thita T., Mungthin M., Dungchai W. (2020). Development of a fluorescent distance-based paper device using loop-mediated isothermal amplification to detect Escherichia coli in urine. Analyst.

[167] Huang Y., Zhang L., Zhang S., Zhao P., Li L., Ge S., Yu J. (2020). Paper-based electrochemiluminescence determination of streptavidin using reticular DNA-functionalized PtCu nanoframes and analyte-triggered DNA walker. Microchim. Acta.

[168] Bender A.T., Sullivan B.P., Zhang J.Y., Juergens D.C., Lillis L., Boyle D.S., Posner J.D. (2021). HIV detection from human serum with paper-based isotachophoretic RNA extraction and reverse transcription recombinase polymerase amplification. Analyst.

[169] Ahn H., Batule B.S., Seok Y., Kim M.-G. (2018). Single-Step Recombinase Polymerase Amplification Assay Based on a Paper Chip for Simultaneous Detection of Multiple Foodborne Pathogens. Anal. Chem..

[170] Nybond S., Réu P., Rhedin S., Svedberg G., Alfvén T., Gantelius J., Svahn H.A. (2018). Adenoviral detection by recombinase polymerase amplification and vertical flow paper microarray. Anal. Bioanal. Chem..

[171] Rani A., Ravindran V.B., Surapaneni A., Shahsavari E., Haleyur N., Mantri N., Ball A.S. (2021). Evaluation and comparison of recombinase polymerase amplification coupled with lateral-flow bioassay for Escherichia coli O157:H7 detection using different genes. Sci. Rep..

[172] Cheung S.F., Yee M.F., Le N.K., Wu B.M., Kamei D.T. (2018). A one-pot, isothermal DNA sample preparation and amplification platform utilizing aqueous two-phase systems. Anal. Bioanal. Chem..

[173] Horst A.L., Rosenbohm J.M., Kolluri N., Hardick J., Gaydos C.A., Cabodi M., Klapperich C.M., Linnes J.C. (2018). A paperfluidic platform to detect Neisseria gonorrhoeae in clinical samples. Biomed. Microdevices.

[174] Tang R., Yang H., Gong Y., Liu Z., Li X., Wen T., Qu Z., Zhang S., Mei Q., Xu F. (2017). Improved Analytical Sensitivity of Lateral Flow Assay using Sponge for HBV Nucleic Acid Detection. Sci. Rep..

[175] Liu H., Xing D., Zhou X. Point of care nucleic acid detection of viable pathogenic bacteria with isothermal RNA amplification based paper biosensor. Proceedings of the Twelfth International Conference on Photonics and Imaging in Biology and Medicine (PIBM 2014).

[176] Kashir J., Yaqinuddin A. (2020). Loop mediated isothermal amplification (LAMP) assays as a rapid diagnostic for COVID-19. Med. Hypotheses.

[177] Wang X., Xiong E., Tian T., Cheng M., Lin W., Wang H., Zhang G., Sun J., Zhou X. (2020). Clustered Regularly Interspaced Short Palindromic Repeats/Cas9-Mediated Lateral Flow Nucleic Acid Assay. ACS Nano.

[178] Mukama O., Wu J., Li Z., Liang Q., Yi Z., Lu X., Liu Y., Liu Y., Hussain M., Makafe G.G. (2020). An ultrasensitive and specific point-of-care CRISPR/Cas12 based lateral flow biosensor for the rapid detection of nucleic acids. Biosens. Bioelectron..

[179] Li Y., Li S., Wang J., Liu G. (2019). CRISPR/Cas Systems towards Next-Generation Biosensing. Trends Biotechnol..

[180] Azhar M., Phutela R., Ansari A.H., Sinha D., Sharma N., Kumar M., Aich M., Sharma S., Singhal K., Lad H. (2020). Rapid, field-deployable nucleobase detection and identification using FnCas9. bioRxiv.

[181] Broughton J.P., Deng X., Yu G., Fasching C.L., Servellita V., Singh J., Miao X., Streithorst J.A., Granados A., Sotomayor-Gonzalez A. (2020). CRISPR–Cas12-based detection of SARS-CoV-2. Nat. Biotechnol..

[182] Gootenberg J.S., Abudayyeh O.O., Lee J.W., Essletzbichler P., Dy A.J., Joung J., Verdine V., Donghia N., Daringer N.M., Freije C.A. (2017). Nucleic acid detection with CRISPR-Cas13a/C2c2. Science.

[183] Jiang X., Lillehoj P.B. (2021). Lateral flow immunochromatographic assay on a single piece of paper. Analyst.

[184] Zadehkafi A., Siavashi M., Asiaei S., Bidgoli M.R. (2019). Simple geometrical modifications for substantial color intensity and detection limit enhancements in lateral-flow immunochromatographic assays. J. Chromatogr. B.

[185] He X., Liu Z., Yang Y., Li L., Wang L., Li A., Qu Z., Xu F. (2019). Sensitivity Enhancement of Nucleic Acid Lateral Flow Assays through a Physical–Chemical Coupling Method: Dissoluble Saline Barriers. ACS Sens..

[186] Zhang Y., Liu X., Wang L., Yang H., Zhang X., Zhu C., Wang W., Yan L., Li B. (2020). Improvement in Detection Limit for Lateral Flow Assay of Biomacromolecules by Test-Zone Pre-enrichment. Sci. Rep..

[187] Tsai T.-T., Huang T.-H., Chen C.-A., Ho N.Y.-J., Chou Y.-J., Chen C.-F. (2018). Development a stacking pad design for enhancing the sensitivity of lateral flow immunoassay. Sci. Rep..

[188] Choi J.R., Liu Z., Hu J., Tang R., Gong Y., Feng S., Ren H., Wen T., Yang H., Qu Z. (2016). Polydimethylsiloxane-Paper Hybrid Lateral Flow Assay for Highly Sensitive Point-of-Care Nucleic Acid Testing. Anal. Chem..

[189] Choi J.R., Yong K.W., Tang R., Gong Y., Wen T., Yang H., Li A., Chia Y.C., Pingguan-Murphy B., Xu F. (2016). Lateral Flow Assay Based on Paper-Hydrogel Hybrid Material for Sensitive Point-of-Care Detection of Dengue Virus. Adv. Health Mater..

[190] Tang R.H., Na Liu L., Zhang S.F., Li A., Li Z. (2019). Modification of a nitrocellulose membrane with cellulose nanofibers for enhanced sensitivity of lateral flow assays: Application to the determination of Staphylococcus aureus. Microchim. Acta.

[191] Yew C.-H.T., Azari P., Choi J.R., Li F., Pingguan-Murphy B. (2018). Electrospin-coating of nitrocellulose membrane enhances sensitivity in nucleic acid-based lateral flow assay. Anal. Chim. Acta.

[192] Sena-Torralba A., Ngo D.B., Parolo C., Hu L., Álvarez-Diduk R., Bergua J.F., Rosati G., Surareungchai W., Merkoçi A. (2020). Lateral flow assay modified with time-delay wax barriers as a sensitivity and signal enhancement strategy. Biosens. Bioelectron..

[193] Katis I.N., He P.J., Eason R.W., Sones C.L. (2018). Improved sensitivity and limit-of-detection of lateral flow devices using spatial constrictions of the flow-path. Biosens. Bioelectron..

[194] Shen M., Chen Y., Zhu Y., Zhao M., Xu Y. (2019). Enhancing the Sensitivity of Lateral Flow Immunoassay by Centrifugation-Assisted Flow Control. Anal. Chem..

[195] Pereira D.Y., Chiu R.Y., Zhang S.C., Wu B.M., Kamei D.T. (2015). Single-step, paper-based concentration and detection of a malaria biomarker. Anal. Chim. Acta.

[196] Tang R., Yang H., Choi J.R., Gong Y., Hu J., Feng S., Pingguan-Murphy B., Mei Q., Xu F. (2016). Improved sensitivity of lateral flow assay using paper-based sample concentration technique. Talanta.

[197] Tang R.H., Li M., Na Liu L., Zhang S.F., Alam N., You M., Ni Y.H., Li Z.D. (2020). Chitosan-modified nitrocellulose membrane for paper-based point-of-care testing. Cellulose.

[198] Yew C.H.T., Azari P., Choi J.R., Muhamad F., Pingguan-Murphy B. (2018). Electrospun Polycaprolactone Nanofibers as a Reaction Membrane for Lateral Flow Assay. Polymers.

[199] Rivas L., Medina-Sánchez M., De La Escosura-Muñiz A., Merkoçi A. (2014). Improving sensitivity of gold nanoparticle-based lateral flow assays by using wax-printed pillars as delay barriers of microfluidics. Lab Chip.

[200] Moghadam B.Y., Connelly K.T., Posner J.D. (2015). Two Orders of Magnitude Improvement in Detection Limit of Lateral Flow Assays Using Isotachophoresis. Anal. Chem..

